# A Proposed Taxonomy to Holistically Classify Employee Mental Health Programs: Qualitative Taxonomy Development Study

**DOI:** 10.2196/67752

**Published:** 2025-12-18

**Authors:** Benedict Sevov, Robin Huettemann, Maximillian Zinner, Sven Meister, Leonard Fehring

**Affiliations:** 1 Faculty of Health, School of Medicine, Witten/Herdecke University, Alfred-Herrhausen-Strasse 50, Witten, 58455, Germany, 49 202 8963708; 2 Health Care Informatics, Faculty of Health, School of Medicine, Witten/Herdecke University, Witten, Germany; 3 Department Healthcare, Fraunhofer Institute for Software and Systems Engineering ISST, Dortmund, Germany; 4 Department of Gastroenterology, Helios University Hospital Wuppertal, Witten/Herdecke University, Wuppertal, Germany

**Keywords:** mental health, digital mental health, workplace mental health, employee well-being, employee mental health programs, employee assistance programs, digital mental health interventions, digital mental health technologies, taxonomy, classification

## Abstract

**Background:**

The number and diversity of employee mental health programs (EMHPs), solutions employers offer to their workforce to improve mental health, have expanded rapidly in recent years, driven by advancements in digital technology and increased global awareness of employee mental health. This dynamic has resulted in a diverse and nontransparent EMHP landscape. While existing taxonomies address specific aspects of mental health programs, a comprehensive taxonomy for classifying EMHPs in more detail remains absent. Establishing such a taxonomy would benefit researchers and practitioners by providing a common standard for categorizing EMHPs and thereby enhance transparency.

**Objective:**

This research aimed to develop and evaluate a comprehensive taxonomy to holistically classify EMHPs, providing a practical and standardized tool for various target groups to categorize, develop, and select EMHPs.

**Methods:**

A thorough taxonomy development process with 4 iterations was applied. The first 2 iterations used conceptual-to-empirical approaches and involved scoping reviews to identify relevant dimensions and characteristics of EMHPs. The latter 2 iterations used empirical-to-conceptual approaches and included semistructured qualitative interviews. The third iteration, involving employee interviews, aimed to identify further dimensions and characteristics of EMHPs to develop the initial taxonomy. During the fourth iteration, 17 international experts were interviewed to refine and validate the initial taxonomy. After the fourth iteration, the taxonomy was evaluated by applying it to 3 real-world EMHPs through a focus group with 5 experts to ensure that all ending conditions and the evaluation goals were met. The interrater reliability was analyzed using the proportion of observed agreement and Fleiss κ.

**Results:**

The resulting taxonomy comprises 2 metadimensions, 21 dimensions, and 69 characteristics, offering a standardized framework for EMHP classification and analysis. Experts successfully applied the taxonomy to classify 3 selected EMHPs, resulting in an overall proportion of observed agreement of 85% and a Fleiss κ of 66%. Across dimensions, the proportion of observed agreement ranged from 64% to 100%, with Fleiss κ ranging from 20% to 100% (*P* values ranging from *P*=.004 to *P*<.001).

**Conclusions:**

This taxonomy contributes to establishing a common standard for holistic EMHP classification. It benefits both mental health researchers and practitioners in fostering transparency and serves as a structured tool for EMHP analysis. The taxonomy enables researchers to conduct relevant future research, including the systematic identification of EMHP archetypes. In practice, the taxonomy can guide providers in market gap identification and EMHP development, inform employers in decision-making, and assist policymakers in setting up targeted support mechanisms for EMHP implementation.

## Introduction

### Background

Deteriorating mental health poses a major global challenge [1], particularly affecting the working population, which represents a substantial share of global society [2,3]. As a result, employee mental health has gained increasing attention from both research and society in recent years [4-6]. Employers are increasingly acknowledging the relevance of employee mental health and providing targeted support. In response, the number and diversity of employee mental health programs (EMHPs)—defined here as solutions employers offer to their employees to sustain or regain good mental health [7]—have expanded rapidly [8-11]. This evolution has been largely driven by digital technology [12-14]. Numerous new players have entered the market with technology-based EMHPs [15], including several apps, platforms, and novel artificial intelligence–enabled tools that address employees’ mental well-being [16].

Transparency on available and examined programs is paramount as researchers investigate different aspects of EMHPs, such as their effectiveness and use patterns [9,17-19]. Metareviews, for example, often focus on specific EMHP types, such as digital ones [12,20,21]. Despite the presence of numerous segmentation criteria, there is still no widely accepted framework with standardized definitions to clearly describe or classify EMHPs. Furthermore, both employer representatives responsible for employee mental health and employees themselves should have access to better resources to understand what programs are available and suitable for their needs [11]. Developing a comprehensive taxonomy to categorize these programs across multiple relevant criteria would enhance transparency, support informed decision-making, and improve accessibility for all stakeholders.

### Prior Work

Currently, few taxonomies exist for classifying mental health programs—particularly EMHPs—and those that do typically address only specific aspects rather than providing a comprehensive, holistic framework. To illustrate, Muñoz et al [22] present a taxonomy to classify mental health interventions by delivery mode. Similarly, Liverpool et al [23] review different digital delivery modes of youth mental health interventions. Gagnon et al [24] and Pineda et al [25] developed taxonomies with 4 and 6 dimensions, respectively, each representing a criterion or parameter, for classifying digital mental health interventions (DMHIs). These taxonomies enabled a more multidimensional approach to programs, moving beyond a single-perspective approach. Other taxonomies include Lukka and Palva’s [26] classification of DMHIs based on gamification levels and the stepped care model of Ferrari et al [27] for classifying video game–based interventions targeting young people. Two additional frameworks have recently been published. Bradley et al [28] propose a classification system to guide users in choosing suitable DMHIs, using a 6-statement decision aid, in which each statement represents a relevant dimension of DMHIs. Hopkin et al [29] offer a framework with 8 dimensions for categorizing digital mental health technologies (DMHTs). Although these 2 taxonomies offer more comprehensive structures, each encompassing more than 5 dimensions, they address general digital programs and do not account for the specific needs of EMHPs. More broadly, existing taxonomies—whether focused on digital or nondigital programs—tend to overlook occupational mental health contexts and fail to consider dimensions particularly relevant for EMHPs. One exception is the work by David et al [30], which presents an approach to occupational mental health for health care workers focusing on distinct mental health stages. This framework is derived from the integrated approach to workplace mental health by LaMontagne et al [31]. However, this approach does not classify EMHPs along several dimensions. The newer taxonomy by Thomas et al [11] specifically addresses EMHPs, classifying them along 3 dimensions: primary purpose, delivery agent location, and latitude in providing. While this taxonomy provides a valuable initial structure for EMHP classification, it lacks the breadth to capture a wider range of relevant dimensions that consider other critical aspects of EMHPs.

### Objectives

To address the limited comprehensiveness of existing taxonomies for EMHPs, we aimed to (1) develop a comprehensive yet concise taxonomy for classifying EMHPs along multiple dimensions, and (2) evaluate the taxonomy by applying it to 3 selected real-world EMHPs.

## Methods

### Study Context

This study forms part of a broader research endeavor by the authors’ research team, which investigates various facets of EMHPs, including employee preferences, use behavior, influencing factors for use, and the overall landscape and types. A clear understanding of EMHP types is essential for conducting transparent and insightful analyses of the aforementioned aspects. Given the lack of a comprehensive taxonomy that holistically classifies EMHPs, we initiated this study to develop such a framework.

### Taxonomy Development Process

**Figure 1.**
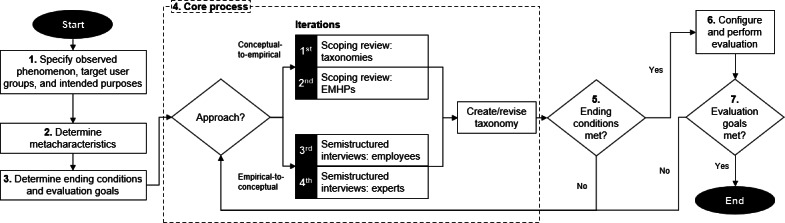
Taxonomy development process according to the recommendations of Nickerson et al [32] and Kundisch et al [33]. EMHPs: employee mental health programs.

We followed the thorough and well-established taxonomy development process outlined by Nickerson et al [32] and the widely adopted extension proposed by Kundisch et al [33] (Figure 1). The process began with the preparation steps, including the definition of metacharacteristics, ending conditions, and evaluation goals. We then conducted the core development process, completing 4 iterations. Finally, we evaluated the resulting taxonomy.

### Preparation Steps

Metacharacteristics represent the foundational components of taxonomy design, including objects, dimensions, and characteristics. To ensure conceptual clarity, we defined the metacharacteristics at the outset of the study. Drawing from the research context, we defined the objects of this taxonomy as all possible EMHPs, the dimensions as the criteria or parameters by which EMHPs can be described and classified, and the characteristics as the specific attributes or options that can be attributed to each dimension. Ending conditions refer to defined states that indicate when the taxonomy development process can be concluded. We defined 5 subjective and 5 objective ending conditions based on the study by Nickerson et al [32] (Table 1).

**Table 1.** Defined subjective and objective ending conditions according to the recommendations of Nickerson et al [32].

Ending condition	Description
Subjective	
Concise	The taxonomy comprises an appropriate number of relevant dimensions to be meaningful while not overwhelming the user with an unnecessarily extensive number of dimensions.
Robust	The dimensions and characteristics of the taxonomy allow for meaningful differentiation between EMHPs^a^, ensuring that these distinctions are relevant for users.
Comprehensive	All relevant EMHPs can be classified using the taxonomy and relevant characteristics of a random sample of real-world EMHPs are reflected in the taxonomy.
Extendable	New dimensions or characteristics can easily be added to the taxonomy to further evolve it if required by landscape dynamics or further research.
Explanatory	The dimensions and characteristics of the taxonomy reveal relevant and understandable information about any EMHP that is classified using the taxonomy.
Objective	
Completeness	At least 1 EMHP is classified under every characteristic of every dimension to ensure that every characteristic of the taxonomy is represented in the real world.
No addition in the last iteration	No new dimensions or characteristics were added in the last iteration (last interview of the fourth iteration); if this were not true, further validation of the last change would be required.
No merge or split in the last iteration	No dimensions or characteristics were merged or split in the last iteration (last interview of the fourth iteration); if this were not true, further validation of the last change would be required.
Uniqueness of dimensions	Every dimension is unique and not repeated, that is, no dimension duplication; otherwise, the taxonomy would contain redundancies.
Uniqueness of characteristics	Every characteristic is unique within its dimension, that is, no characteristic duplication within 1 dimension; otherwise, the taxonomy would contain redundancies.

a EMHP: employee mental health program.

Evaluation goals refer to specific activities a finalized taxonomy should be able to perform. We defined 3 evaluation goals in line with our research objectives and based on the study by Kundisch et al [33] (Table 2).

**Table 2.** Defined evaluation goals according to the recommendations of Kundisch et al [33].

Evaluation goal	Description
Describing	The taxonomy can be used to describe EMHPs^a^ by several characteristics across multiple dimensions, meaning users can present relevant information about any EMHP using the taxonomy.
Classifying	The taxonomy can be used to classify EMHPs by assigning at least 1 characteristic per each dimension, meaning users can comprehensively describe any EMHP, presenting information for every dimension of the taxonomy.
Analyzing	The taxonomy can be used to analyze EMHPs based on similarities or differences to other EMHPs across dimensions and characteristics, meaning users can compare different EMHPs with each other and detect the most relevant similarities or differences.

a EMHPs: employee mental health programs.

### Core Taxonomy Development Process

#### Overview

The first 2 iterations of the core taxonomy development process used conceptual-to-empirical approaches, involving 2 scoping reviews according to the PRISMA-ScR (Preferred Reporting Items for Systematic Reviews and Meta-Analyses Extension for Scoping Reviews) guidelines [34] to generate first codes for the initial taxonomy. The final 2 iterations used empirical-to-conceptual approaches with semistructured qualitative interviews to identify additional codes and to refine and validate the taxonomy.

#### First Iteration

The first scoping review (first iteration) aimed to identify relevant taxonomies on general mental health programs, including EMHPs, and was conducted on January 2, 2024, using the following 2 search queries on PubMed:

1. ((taxonomy[MeSH Terms]) OR (framework[MeSH Terms]) OR (structure[MeSH Terms]) OR (classification[MeSH Terms]) OR (categorization[MeSH Terms])) AND ((mental health intervention[MeSH Terms]) OR (mental health program[MeSH Terms]))
2. ((taxonomy[Title/Abstract]) OR (framework[Title/Abstract]) OR (structure[Title/Abstract]) OR (classification[Title/Abstract]) OR (categorization[Title/Abstract])) AND ((mental health intervention*[Title/Abstract]) OR (mental health program*[Title/Abstract]))

The first review’s PRISMA-ScR checklist [34], detailed screening process (Multimedia Appendix 1), and overview of the literature records (Multimedia Appendix 2) are provided for further details.

#### Second Iteration

The second scoping review (second iteration) aimed to identify relevant studies specifically on EMHPs and was initially conducted on January 12, 2023, with the literature records having been analyzed in January 2024, using the following 2 search queries on PubMed:

1. ((mental health[MeSH Terms]) OR (psychology[MeSH Terms])) AND ((employee assistance program[MeSH Terms]) OR (employer intervention[MeSH Terms]) OR (workplace intervention[MeSH Terms])) OR ((mental health[MeSH Terms]) AND (workplace[MeSH Terms]) AND (health promotion[MeSH Terms]))
2. (mental health[Title/Abstract]) AND ((employee assistance program[Title/Abstract]) OR (employer intervention[Title/Abstract]) OR (workplace intervention[Title/Abstract]))

For both reviews (first and second iterations), we followed established procedures to identify the respective search terms. We conducted initial paper searches to identify relevant papers and derive established and widely used keywords and MeSH terms. We subsequently discussed these terms within our entire author team—including SM and LF, who have extensive experience conducting scoping reviews and have published more than 20 peer-reviewed papers—prior to executing the final searches. In 2 coding rounds, authors BS and RH systematically screened the resulting papers from both reviews and assigned codes to text segments representing potential dimensions and characteristics of EMHPs. The final set of codes informed the initial taxonomy. For the second review, similar documentation is provided as for the first (MultimediaAppendices 3  4), detailing how codes were derived and incorporated into the taxonomy.

#### Third Iteration

During the third iteration, we conducted interviews with a representative sample of employees, selected to reflect diversity across characteristics, such as age, gender, and education. We recruited the participants through purposive sampling from the broader network of all authors to ensure representativeness. These interviews were conducted from February to March 2023 via phone or video calls or in person and contributed to 2 research studies conducted by our research team, while only 1 part of the factually anonymized transcripts was analyzed for this study. For this study, the purpose of the interviews was to identify relevant dimensions and characteristics of EMHPs to inform the initial taxonomy, whereas for the other study, the purpose was to identify relevant EMHP types, as well as facilitators of and barriers to using EMHPs. The findings of the other study were published in a peer-reviewed paper on employee preference and use behavior by Sevov et al [7]. The EMHP dimensions and characteristics for this study were derived based on thematic analysis according to Braun and Clarke [35], which was conducted in 2 coding rounds by authors BS and RH. To ensure transparency and methodological rigor, we provide additional details, including the interview guide (Multimedia Appendix 5), the COREQ (Consolidated Criteria for Reporting Qualitative Research) checklist [36], and an overview of the participant sample (Multimedia Appendix 6).

#### Fourth Iteration

During the fourth iteration, we conducted interviews with employee mental health experts to refine and validate the initial taxonomy. We recruited experts from 3 groups: first, relevant representatives from employers offering EMHPs (referred to as employers); second, representatives from providers of EMHPs (providers); and third, academic key opinion leaders (KOLs) who conduct research in employee mental health and related fields. To ensure geographic and cultural diversity, we systematically recruited employers and providers from multiple countries, including Germany, the United Kingdom, France, the United States, and Japan. Employers were identified through LinkedIn keyword search per country (“employee mental health” and “occupational mental health”) and were contacted directly. Providers were identified through Google keyword searches in English per country (“employee mental health program provider COUNTRY”), and representatives were contacted using publicly available information. For Germany and France, these searches were additionally conducted in the respective local languages. KOLs were selected based on publication volume in employee mental health research, identified through a PubMed keyword search (“(employee mental health[Title/Abstract]) OR (employee mental health program[Title/Abstract]) OR (employee assistance program[Title/Abstract])”), and were contacted using publicly available email addresses. In total, we contacted 73 experts: 43 employers, 20 providers, and 10 KOLs.

These interviews were conducted from January to April 2024 via video calls and involved presenting and discussing the taxonomy. Using an iterative approach, we revised the taxonomy after each interview based on expert feedback and presented the updated version to the next interviewee. This process continued until both the predefined ending conditions and the minimum requirements for a representative expert sample were met, concluding after the 17th interview. The final expert sample included 5 participants from Germany, 3 from the United States, 3 from the United Kingdom, 2 from France, 2 from Japan, 1 from Sweden, and 1 from Mexico. Of these, 8 were employers, 5 were providers, and 4 were KOLs. Following the final interview, we reviewed all transcripts within the author team and made final adjustments to the taxonomy on wording based on the interview inputs to ensure clarity and consistency. Additional details related to this iteration are provided, including the interview guide (Multimedia Appendix 7), the COREQ checklist [36], and the participant sample (Multimedia Appendix 8).

### Taxonomy Evaluation

#### Evaluation Design

**Figure 2.**
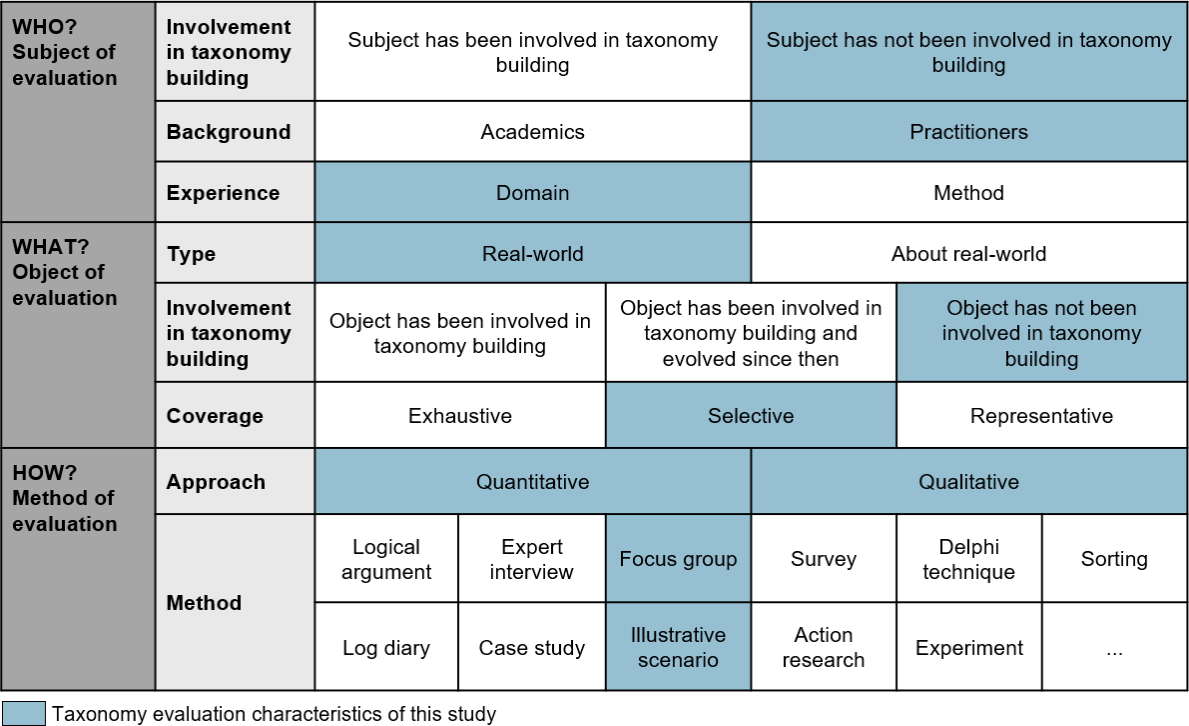
Applied taxonomy evaluation design based on the framework for taxonomy evaluation from the study by Szopinski et al [37].

After the fourth iteration, we conducted a focus group to evaluate the taxonomy—a well-established method for taxonomy evaluation [37]. Using the 3 predefined evaluation goals, 5 experts participated in the evaluation, applying the taxonomy to 3 established real-world EMHPs. The evaluation design was carefully defined based on the framework for taxonomy evaluation proposed by Szopinski et al (Figure 2) [37].

#### EMHP Selection

The selected EMHPs were established programs that had been available on the market for at least 3 years. This means that they were publicly available, for example, via a website, generally recognized, that is, among the top results in online or app store searches, and adopted and offered by multiple organizations, as evidenced by client listings on provider websites. Importantly, none of the selected EMHPs were affiliated with experts from the fourth iteration. We also selected these programs to ensure diversity: one was a fully digital online platform for employee mental health, another a purely app-based mindfulness program, and the third a program that featured digital and nondigital components focused on coaching and counseling.

#### Expert Selection

The experts were identified through purposive sampling to ensure that all participants were EMHP specialists with practitioner background and none of them had been involved in the fourth iteration. The selected EMHP experts represented diverse educational backgrounds, primarily in psychology or business economics, with several holding a PhD and having experience in scientific research. To ensure adequate program-specific insight, 1 representative from each of the 3 classified EMHPs participated in the focus group. During the moderated session, the experts first reflected on the taxonomy’s robustness, comprehensibility, and wording. They then independently applied the taxonomy to 1 of the 3 real-world EMHPs by selecting relevant characteristics per dimension, which was followed by a group discussion on the taxonomy and the first classification [37]. After the focus group, the same experts were asked to apply the taxonomy to the remaining 2 EMHPs in written form to ensure independent and unbiased classification.

#### Qualitative Analysis

The qualitative reflection of the taxonomy during the focus group comprised explicit expert input on the subjective ending conditions and the understandability of the taxonomy. The experts debated whether more or less dimensions or characteristics should have been added and whether the presented characteristics were meaningful and clear to users. They weighed the advantages and disadvantages of possible changes and provided explicit and consensual conclusions on these potential adjustments. Authors BS and LF conducted the focus group and reviewed the field notes and the transcript after the session to qualitatively analyze whether the subjective ending conditions were met based on explicit expert statements and whether the experts were able to use the taxonomy to classify EMHPs based on the classification of the first EMHP and the subsequent group discussion.

#### Interrater Analysis

We assessed interrater reliability among the focus group experts regarding their classifications using the proportion of observed agreement and Fleiss κ [38]. We used Fleiss’ approach as it represents an established extension of Cohen κ that can be applied when having a constant number of more than 2 raters assessing nominal or categorical variables, which was the case in our taxonomy evaluation [38,39]. Characteristics were coded as binary variables, that is, 1=selected, 0=not selected, to meet the mutual exclusivity condition required for Fleiss κ analysis [40]. The proportion of observed agreement and κ were calculated for each dimension. We then computed the unweighted average across dimensions to determine the overall proportion of observed agreement and overall κ. This analysis provided empirical data that complemented the qualitative focus group discussions and supported the conclusion of the taxonomy evaluation against the predefined evaluation goals. Additional details of the evaluation process are provided, including the COREQ checklist [36] and the ACCORD (Accurate Consensus Reporting Document) checklist [41].

### Ethical Considerations

The ethics committee of Witten/Herdecke University reviewed and approved this research project without raising any ethical concerns (S-317/2023). Voluntary and informed consent was obtained in advance and in writing from all participants of the qualitative interview studies and the focus group, covering both participation and publication of study findings in a peer-reviewed paper. The interview studies and the focus group adhered to established ethical scientific research standards and complied with the General Data Protection Regulation. The recordings of the qualitative interviews and the focus group session were transcribed in a factually anonymized way, ensuring that individual participants could not be identified—or only with disproportionate effort [42]. The participants of the interview studies and the focus group received no monetary compensation for their involvement.

## Results

### Final Taxonomy

**Figure 3.**
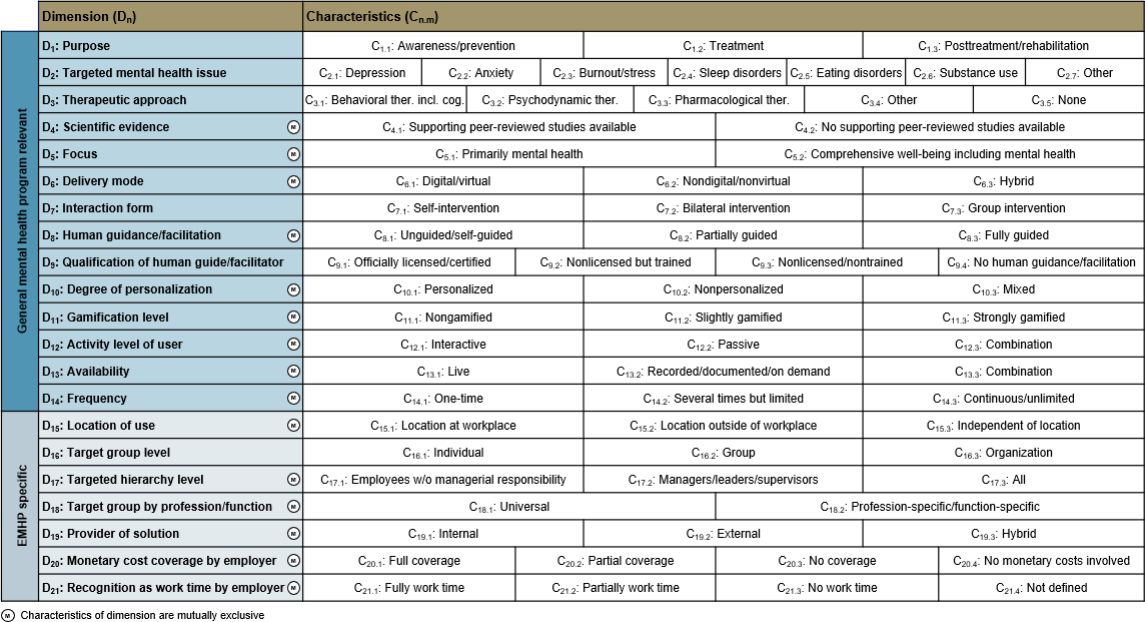
Final taxonomy to classify employee mental health programs.

**Figure 4.**
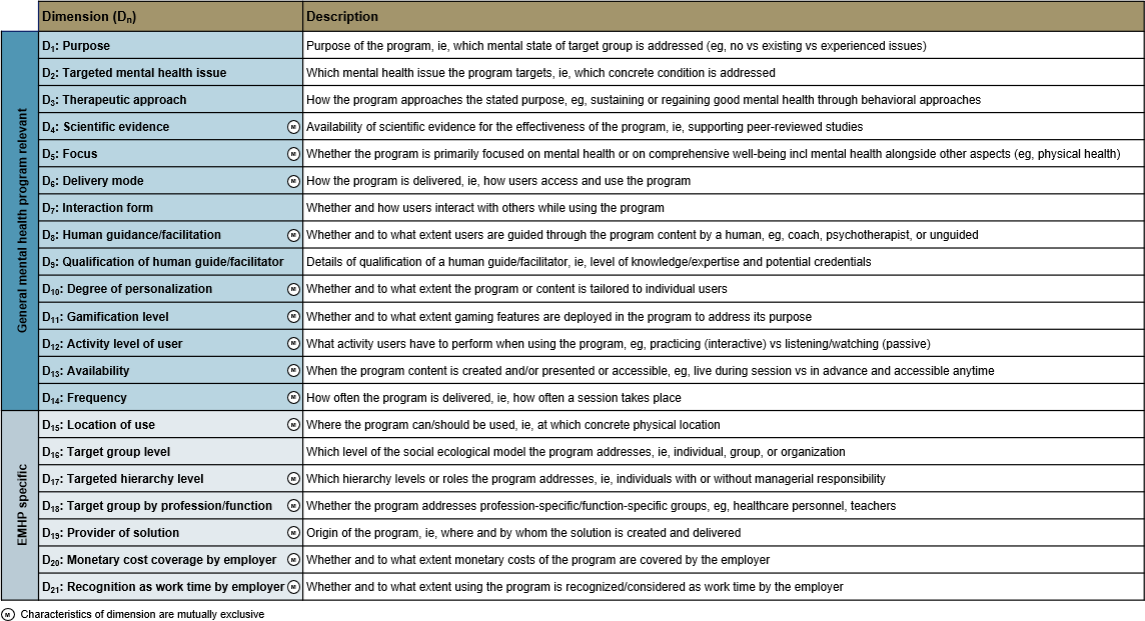
Descriptions of all dimensions of the final employee mental health program taxonomy.

The final taxonomy is presented as a morphological box comprising 2 metadimensions, that is, distinct categories of dimensions, 21 dimensions (D_n_) and 69 characteristics (C_n.m_) (Figure 3). A morphological box is defined as “a structured method for systematically looking at key characteristics or parameters of a solution [ie, dimensions] and the realistic options for each parameter [ie, characteristics]” [43]. This format offers an intuitive and systematic way to visualize the taxonomy, allowing users to mark applicable characteristics of any EMHP and generate a visual classification. To avoid misinterpretation, all dimensions are described in detail (Figure 4). The first metadimension (D_1_-D_14_) applies to all mental health programs, while the second one (D_15_-D_21_) captures EMHP-specific aspects. Classification across dimensions is based on the nature of the EMHP, that is, the specific program or module being classified, with several dimensions particularly relevant for differentiating digital EMHPs. Although many characteristics within these dimensions (eg, D_10_, D_11_, D_13_, and D_15_) predominantly apply to digital EMHPs, they are not exclusive to them. Following Nickerson et al [32], the taxonomy includes dimensions with mutually exclusive characteristics, meaning only 1 characteristic can apply per dimension. However, certain dimensions (D_1_, D_2_, D_3_, D_7_, D_9_, and D_16_) are not mutually exclusive, allowing multiple characteristics to be selected simultaneously to avoid loss of information and to account for the complex nature of EMHPs [44]. For example, many EMHPs address multiple mental health issues, and it may be particularly valuable to capture the specific combination of issues targeted.

### Dimensions and Characteristics of the Taxonomy

Each iteration of the taxonomy development process contributed to the final taxonomy (Table 3). The first 3 iterations independently yielded relevant dimensions and characteristics that formed the initial taxonomy. The fourth iteration then refined the initial taxonomy and provided valuable insights. While 2 new dimensions were added, the experts consistently confirmed most dimensions and their related characteristics of the initial taxonomy. Only the wording was refined or minor changes, such as adding 1 characteristic, were made and validated during the subsequent interviews. As some dimensions sparked debates, we examined the different expert views and relied on consensus and fit with our research objectives to finally design the respective dimension.

**Table 3.** Summary of results from each of the 4 iterations.

Iteration	Key results
First iteration—scoping review: taxonomies	17 studies presenting relevant underlying taxonomies or frameworks 6 taxonomies or frameworks comprising more than 2 dimensions Only 2 taxonomies or frameworks focusing on the occupational context 14 of the final 21 dimensions derived from this iteration 36 of the final 69 characteristics derived from this iteration
Second iteration—scoping review: EMHPs	37 relevant studies on EMHPs^a^ 18 of the final 21 dimensions derived from this iteration 48 of the final 69 characteristics derived from this iteration 5 new dimensions derived that were not identified in the first iteration 13 dimensions confirmed from the first iteration
Third iteration—semistructured interviews: employees	15 interviews with employees 18 of the final 21 dimensions derived from this iteration 43 of the final 69 characteristics derived from this iteration 18 dimensions from the first and second iterations confirmed, just 1 not No new dimension derived from this iteration
Fourth iteration—semistructured interviews: experts	17 interviews with experts 2 new dimensions added during this iteration 13 new characteristics added during this iteration, of which 6 for the 2 new dimensions 7 dimensions renamed during this iteration 27 characteristics renamed during this iteration

a EMHPs: employee mental health programs.

All 21 dimensions of the final taxonomy are presented in the following, each accompanied by a concise explanation and, where relevant, insights from the expert interviews.

The Purpose (D_1_) of an EMHP defines the program’s core objective. Experts debated how the characteristics reflect programs addressing chronic mental health conditions. Based on expert consensus, active management of chronic mental illness was categorized under Treatment (C_1.2_), since such conditions are typically treated regardless of the temporary severity and the frequency and duration of formal treatment. The characteristics of this dimension are therefore based on the mental health stage of the individual, representing a framework that is both concise and established in the literature [10,17,45].

The Targeted mental health issue (D_2_) specifies which concrete conditions an EMHP addresses. Experts discussed whether to define issues by symptoms or diagnoses. We purposely focused on diagnosable conditions according to the *International Classification of Diseases, Eleventh Revision* (*ICD-11*) by the World Health Organization while also including certain symptoms explicitly listed in *ICD-11* when particularly relevant [46]. Several experts suggested including other issues as characteristics. This represented a trade-off between robustness and conciseness, as we targeted meaningful differentiation without listing too many characteristics. While no explicit cutoff point was defined, we prioritized the characteristics presented in the final taxonomy based on the frequency of mental health issues targeted in existing EMHPs and the input from literature and the experts. Besides, geographic variations in relevance of targeted mental health issues emerged from the expert interviews: in Japan, for example, substance use was considered seldom while anger management was rated important, and in the United States, grief and loss, suicidal ideation, and neurodivergence were considered more relevant. Ultimately, these mental health issues were categorized under Other based on their lower prevalence in the analyzed EMHPs and the consolidated expert input.

The Therapeutic approach (D_3_) indicates whether an EMHP is based on established therapeutic approaches, that is, clearly defined and well-researched approaches. Experts agreed on including Behavioral (C_3.1_), Psychodynamic (C_3.2_), and Pharmacological therapy (C_3.3_), confirming cognitive behavior therapy as the most prevalent in occupational contexts, consistent with metareview findings [10,17,21,45]. As with D_2_, for D_3_, several other examples were stated by the experts. While single experts argued for the inclusion of holistic therapy, systemic therapy, hypnotherapy, and other concrete examples, these less prevalent approaches were categorized under Other (C_3.4_). We also considered whether coaching—or related approaches such as counseling or consulting—should be included as its own therapeutic approach. We chose not to include this characteristic in the initial taxonomy, as coaching, counseling, or consulting encompasses a wide range of formats and does not represent clearly defined therapeutic approaches. Instead, coaches may draw on elements from various established therapeutic approaches in their coaching sessions, which the taxonomy can accommodate. For example, if a program offers coaching that is explicitly based on behavioral therapy, the characteristic Behavioral therapy including cognitive (C_3.1_) can be selected. In addition to the included approaches, we added the characteristic None (C_3.5_) to capture EMHPs that provide information or raise awareness on employee mental health without a therapeutic approach, for example, through leaflets, books, or awareness sessions. This refinement was proposed by one expert and subsequently validated by all other experts.

The Scientific evidence (D_4_) captures whether peer-reviewed studies exist for the specific EMHP, focusing on the program itself rather than its underlying therapeutic approach, since delivery can influence effectiveness. The Focus (D_5_) distinguishes between EMHPs that concentrate primarily on mental health and those that address broader aspects of employee well-being, such as physical health alongside mental health. The Delivery mode (D_6_) describes how users access and use the program, particularly whether digital components are involved. The Interaction form (D_7_) specifies whether and how EMHP users interact with others during program use. The Human guidance/facilitation (D_8_) indicates whether and to what extent EMHP use is guided by a human. The Qualification of human guide/facilitator (D_9_) details the credentials of a human guide, such as officially approved licenses and certifications or professional training programs, which determine the reliability and suitability of an EMHP. The Degree of personalization (D_10_) reflects the extent to which EMHP content or delivery is tailored to individual users. The Gamification level (D_11_) indicates the extent to which gaming features are present in an EMHP. Although one expert raised concerns about this dimension, suggesting that gamification might trivialize mental health issues and undermine program objectives, the dimension was retained based on consensus among other experts, the growing use of gamified EMHPs [26,27], and evidence indicating that gamification can lead to higher intention to use, particularly among men [19]. The Activity level of user (D_12_) describes the degree of activity required when using an EMHP. The Availability (D_13_) specifies whether EMHP content is accessible on demand or bound to fixed times. The Frequency (D_14_) captures how often an EMHP is delivered. The Location of use (D_15_) identifies where an EMHP is used, such as the workplace or externally. Although one expert questioned this dimension’s relevance, both previous research and the employee interviews suggest that the location can influence EMHP use rates due to stigmatization and anonymity concerns [47]. The Target group level (D_16_) specifies the level of the social ecological model [48], meaning whether an EMHP targets individual employees, groups such as departments and project teams, or entire organizations. Organization-wide antistigma awareness programs are a prominent example of the latter [49]. The Targeted hierarchy level (D_17_) describes which employees are addressed by formal role, with many EMHPs specifically targeting managers or executives, who are all subsumed under C_17.2_. The Target group by profession/function (D_18_) indicates whether an EMHP is tailored to specific professions or functions. The Provider of solution (D_19_) identifies who develops or delivers an EMHP. The Monetary cost coverage by employer (D_20_) specifies whether and to what extent the employer covers the monetary costs of an EMHP. The Recognition as work time by employer (D_21_) indicates whether participation in an EMHP is counted as work time. This dimension was added based on one expert’s view suggesting that this strongly influences use. For both D_20_ and D_21_, the defining characteristics are shaped by the employer offering the EMHP rather than the program itself. Nonetheless, both dimensions were included because experts and literature highlight their relevance for use [50], and many EMHPs predefine a single characteristic by design.

### Application and Evaluation of the Taxonomy

The taxonomy was evaluated through the focus group with 5 experts, which generated valuable qualitative insights from the discussion and quantitative empirical data from the interrater analysis of the classifications of the 3 established German EMHPs: the holistic digital mental health platform “Likeminded,” the mindfulness mobile app “7Mind,” and the “Coaching & Counseling” service from the “Fuerstenberg Institut.”

At the beginning of the focus group session, the experts reflected on the taxonomy and confirmed that it provides an appropriate overview of relevant EMHP dimensions and a meaningful level of detail for analysis. The reflection further indicated that the balance between comprehensiveness and conciseness was well achieved. It also showed that users of the taxonomy need to familiarize themselves with the dimension descriptions to ensure a consistent understanding. The discussion of the first classification further demonstrated that reliable use of the taxonomy requires both a solid understanding of its dimensions and sufficient knowledge of the programs being classified.

The interrater analysis yielded a high overall proportion of observed agreement of 85%, interpreted according to Graham et al [51], and indicated a substantial strength of agreement with a κ of 66%, interpreted following Landis and Koch [52]. Across dimensions, the proportion of observed agreement ranged from 64% to 100% and κ ranged from 20% to 100%. The results were statistically significant, with *P* values ranging from *P*=.004 to *P*<.001.

Based on the qualitative reflections and the successful classification of the 3 EMHPs with high overall agreement measures, we concluded that the taxonomy was concise, robust, comprehensive, extendible, and explanatory, which reconfirmed that the defined subjective ending conditions were met [32]. The successful classifications also demonstrated that the taxonomy can be applied to describe, classify, and analyze EMHPs, fulfilling the predefined evaluation goals [37]. Additional details on the evaluation results, including the 3 classifications and a table with the key statistical measures of the interrater analysis per dimension, are provided (Multimedia Appendix 9).

## Discussion

### Principal Findings

No taxonomy is ever definitive [44] or can comprehensively capture all possible dimensions and characteristics due to the inherent complexity and evolving nature of EMHPs. Nonetheless, our proposed taxonomy provides a robust and comprehensive framework for holistically classifying the existing and emerging landscape of EMHPs, while remaining generally extendible, which is an important aspect of a useful taxonomy [32]. To achieve this, we carefully considered trade-offs in the inclusion of dimensions and characteristics, especially between comprehensiveness, robustness, and conciseness [32]. The high overall proportion of observed agreement and the substantial interrater reliability achieved during evaluation [51,52] corroborate the robustness of our taxonomy.

Variation in the proportion of observed agreement and κ across dimensions can be partly attributed to the number of available characteristics and whether they are mutually exclusive. This is exemplified by the dimension Targeted mental health issue (D_2_), which yielded the lowest proportion of observed agreement, with 64%, and the lowest κ, with 20%, and has the highest number of characteristics, that is, 7, which are not mutually exclusive. While this dimension did not lead to a fundamental discussion among focus group experts, the results indicated that experts have very subjective views on which mental health issues are addressed by a program. When multiple characteristics can be selected, classification outcomes are more likely to diverge, especially when users have varying levels of familiarity with the EMPH being assessed. Accordingly, user knowledge of an EMHP is critical for producing reliable classifications. Users should thoroughly familiarize themselves with a program before applying the taxonomy to ensure accurate and consistent results. In parallel, EMHP providers should clearly communicate the intended scope and design of their programs to support stakeholder understanding.

To ensure the comprehensiveness of our taxonomy, we included all relevant dimensions without exceeding a reasonable number. In doing so, we referred to other published taxonomies with relatively many dimensions, for example, Denecke and May [53], Scheider et al [54], and Weking et al [55] with 18, 18, and 22, respectively. We deliberately grounded our taxonomy in existing research by incorporating relevant frameworks, including the DMHI taxonomy of Gagnon et al [24], the game-based DMHI categories by Lukka and Palva [26], and the *ICD-11* by the World Health Organization [46].

Compared with other existing taxonomies, our taxonomy offers a comprehensive structure specifically designed to classify EMHPs and stands out in 2 key ways. First, with the exception of the taxonomies by Thomas et al [11] with 3 dimensions and David et al [30] and LaMontagne et al [31] with 1 dimension each, ours is to the best of our knowledge the only taxonomy explicitly developed for EMHPs rather than for general or other mental health programs. Second, while these other 3 taxonomies comprise 1-3 dimensions, our taxonomy is the only one to capture a broader and more detailed range of EMHP dimensions relevant to both academics and practitioners. This does not diminish the value and purpose of the existing taxonomies, many of which are intentionally focused on specific program scopes, dimensions, or both. The selection of an appropriate taxonomy ultimately depends on the user’s objective; more targeted taxonomies may be preferable when examining a specific aspect of EMHPs or general mental health programs. However, we argue that our taxonomy encompasses most of the dimensions found in other frameworks—particularly those with only 1 or 2 dimensions—as we systematically reviewed and incorporated all relevant taxonomies and frameworks identified during the first iteration of our taxonomy development process. To demonstrate the unique value proposition of our taxonomy, we compared it with other taxonomies with more than 2 dimensions in terms of scope and value proposition (Table 4). In this comparison, we also included taxonomies published after concluding but before publishing our study. We did not compare taxonomies with 1 or 2 dimensions in more detail, as these do, by definition, not represent comprehensive taxonomies. Notably, most of the taxonomies we identified and reviewed have been published within the past 5 years.

**Table 4.** Overview of relevant taxonomies on mental health programs with more than 2 dimensions and their respective scope and value proposition.

Study	Purpose	Program scope	Focus on EMHPs^a^	Number of dimensions	Key dimensions	Value proposition
This study						
EMHP taxonomy	Providing a comprehensive taxonomy to holistically classify EMHPs	EMHPs	Yes	21	Purpose Therapeutic approach Delivery mode Interaction form Frequency Target group level Provider of solution Monetary cost coverage by employer	Comprehensive taxonomy on EMHPs covering multiple dimensions across two metadimensions: (1) General mental health program relevant, and (2) EMHP specific, thereby consolidating various dimensions from existing taxonomies
Scoping review 1: search						
Burger et al (2020) [10]	Providing an overview of the system landscape of electronic mental health interventions for depression to differentiate programs by system type	Electronic mental health interventions for depression	No	9	Technology Function type Purpose Therapy class Quality	Overview covering dimensions describing digital mental health programs across relevant criteria and also presenting the evaluation methods used
Clay et al (2020) [56]	Providing a framework to identify and categorize core components of mental health stigma interventions in LMICs^b^	Mental health stigma reduction interventions in LMICs	No	13	Target population Qualifications of delivery agents Content detail Duration Outcome assessments Intervention methods Dissemination	Comprehensive framework covering digital and nondigital mental health programs in LMICs identified in scientific studies
Gagnon et al (2022) [24]	Providing a framework to classify DMHIs^c^	DMHIs	No	4	System Function Time Facilitation	Targeted framework classifying DMHIs focusing on technology and delivery method
Lukka and Palva (2023) [26]	Providing a framework to develop game-based DMHIs	Game-based DMHIs	No	4	Target audience Engagement Effectiveness	Framework supporting the development of game-based DMHIs, focusing on combining health care delivery and entertainment
Pineda et al (2023) [25]	Providing an updated taxonomy to categorize literature on and different types of DMHIs for treatment and prevention	DMHIs	No	6	The taxonomy presents 6 parameters, that is, dimensions, of DMHIs, which are not labeled; the first dimension presents 4 types of DMHIs: Provider administered Provider administered with blended digital adjuncts Self-help human supported or guided Self-help fully automated	Updated taxonomy categorizing the literature on DMHIs for treatment and prevention
Scoping review 1: snowballing						
Chan et al (2015) [57]	Providing input on a possible framework to evaluate mobile mental health apps	Mobile mental health apps	No	3	All 3 dimensions with 4-5 subdimensions; Usefulness Usability Integration and infrastructure	Proposed components of a framework for finding and evaluating mobile mental health apps that can be used by patients and providers
Relevant new taxonomies published after conclusion of this study						
Bradley et al (2025) [28]	Providing a classification system to support users in deciding on a suitable DMHI	DMHIs	No	6	Target population Function System Cost and access	Classification system as decision aid empowering users to make an informed choice for a suitable DMHI based on preference
Hopkin et al (2025) [29]	Providing a framework to categorize DMHTs^d^	DMHTs	No	8	Population Purpose Type of approach Human interaction Functionality	Framework designed for various stakeholders for categorizing DMHTs and identifying key characteristics of DMHTs
Thomas et al (2024) [11]	Providing a taxonomy to organize and distinguish workplace mental health offerings	Workplace mental health offerings	Yes	3	Purpose Delivery agent Latitude in providing	Taxonomy for organizing workplace mental health offerings along 3 dimensions

a EMHPs: employee mental health programs.

b LMICs: low- and middle-income countries.

c DMHIs: digital mental health interventions.

d DMHTs: digital mental health technologies.

### Theoretical and Practical Implications

Our comprehensive taxonomy offers valuable contributions to both research and practice. For researchers, it provides a standardized framework for classifying EMHPs, facilitating metareviews or effectiveness assessments, as in Carolan et al [20], and for analyzing program limitations, such as the role of human guidance in digital EMHPs [47].

Practitioners also benefit from its structured approach. Providers can identify market gaps or refine their offerings based on EMHP archetypes, for example, derived from the 4 types of DMHIs proposed by Muñoz et al [22]. Developers may use the taxonomy to design more targeted and effective programs [30], while employers can apply it to guide decisions about which EMHPs to offer. Key considerations—such as whether mental health support is offered internally or through an external provider, and the associated cost [9,58]—can be examined along the taxonomy’s individual dimensions. Employers can also use the taxonomy to align their EMHP selection with the characteristics of their workforce. For example, some professions are dominated by women or men, some organizations employ highly educated professionals, and others have predominantly young employees. Existing research offers insights into how sociodemographic employee groups differ in their preferences for EMHP features [7,19]. As an example, employers can use the taxonomy to design and conduct internal surveys to assess their employees’ EMHP needs and preferences.

Given their potential to promote mental well-being and contribute to a mentally healthier workforce—and by extension, a healthier society—employers should be encouraged to offer EMHPs, regardless of national legislative requirements. The taxonomy is intended to support more informed and accessible decision-making for employers, while also serving as a resource for policymakers to develop targeted support mechanisms and recommendations for EMHP implementation.

### Future Research

The aim of our taxonomy was to contribute to both research and the implementation of EMHPs across all types of employers, including small- and medium-sized enterprises, large corporations, and other employer types such as public organizations. Accordingly, ensuring universal applicability was a key objective, positioning the taxonomy as a potential new standard. Beyond its immediate use, the taxonomy also serves as an appropriate starting point for future research and targeted adaptations for specific application contexts.

One possibility for adaptation involves tailoring the taxonomy to specific geographic or cultural settings. Our findings indicate that the prevalence of mental health issues and the therapeutic approaches used vary across cultures. Moreover, the classification of EMHPs is influenced by cultural context. For example, the dimension Qualification of human guide/facilitator may depend on national regulatory frameworks: while some countries enforce strict certification requirements for mental health professionals, others apply more flexible standards and require lower levels of formal training [59,60]. Given the well-documented importance and effectiveness of cultural adaptation in mental health program development and implementation [61-63], we conclude that adapting taxonomies to cultural contexts is both relevant and advisable. Another possibility for adaptation is the refinement of the taxonomy to capture the role of digital technologies in EMHPs in more detail. As digital or hybrid EMHPs become increasingly prevalent [12-14], additional dimensions could be introduced to reflect the technologies used, such as AI-based systems. With the growing integration of AI-based technology in mental health care [64,65], it may be valuable to explicitly capture whether and how AI is used within EMHPs, potentially offering higher granularity.

We also see potential for the taxonomy to support future research aimed at identifying EMHP archetypes. While our evaluation revealed notable differences among the classified programs, further studies classifying a larger number of EMHPs, for example, approximately 30 objects [44], are needed to uncover consistent patterns and derive robust EMHP archetypes. Exploring the typology of digital EMHPs may be particularly relevant given the rapid emergence and evolution of new digital programs.

### Limitations

Despite the thorough taxonomy development process and adherence to established research standards, this study has some typical limitations. First, the taxonomy is based on available literature and subjective expert input, requiring a balance between comprehensiveness and conciseness. As with any taxonomy, one could argue that certain dimensions or characteristics are missing or that some included elements may be less relevant. To mitigate this, we consulted a geographically diverse group of experts, ensuring broad representation and minimizing bias.

Second, although the fourth iteration included experts from 3 distinct groups and 7 countries across 3 continents, 5 of the 17 experts were from Germany. This may have introduced a geographic bias in the validation process, potentially making the taxonomy slightly more applicable for German or European EMHPs. For example, the characteristics included under Targeted mental health issue may reflect regional priorities. A taxonomy designed for other regions of the world might have included a partially different set of mental health issues, such as grief and loss, suicidal ideation, and neurodivergence in North America or anger management in parts of Asia. However, we intentionally chose not to account for country-specific systematic or institutional particularities in the development of our taxonomy. Instead, we relied on global literature and input from geographically diverse experts to determine the most relevant characteristics. We therefore remain confident in the taxonomy’s universal applicability.

Third, although we conducted thorough scoping reviews, it is possible that some relevant publications were missed or that new taxonomies emerged after our research was completed, as was the case with Thomas et al [11]. To enhance overall transparency, we provide a positionality statement on our research (Multimedia Appendix 10).

### Conclusions

This research contributes to establishing a new common standard for the holistic classification of EMHPs. To the best of our knowledge, the proposed taxonomy represents the first comprehensive and in-depth conceptual framework for systematically investigating the evolving landscape of EMHPs at a universal level. As such, the taxonomy offers value to both researchers and practitioners by fostering transparency and providing a shared foundation for analysis. Future research can build on this taxonomy to improve comparability across studies and support the identification of EMHP archetypes through analyses and comparisons of classifications. In practice, program developers and employers may use the taxonomy as a decision-making tool to guide program design, selection, and implementation, while policymakers may leverage it for setting up support mechanisms for EMHP implementation.

## Supplementary material

10.2196/67752 Multimedia Appendix 1 Screening process and final set of literature records of the first iteration.

10.2196/67752 Multimedia Appendix 2 Overview of final set of literature records of the first iteration.

10.2196/67752 Multimedia Appendix 3 Screening process and final set of literature records of the second iteration.

10.2196/67752 Multimedia Appendix 4 Overview of final set of literature records of the second iteration.

10.2196/67752 Multimedia Appendix 5 Relevant part of the interview guide for interviews of the third iteration.

10.2196/67752 Multimedia Appendix 6 Sample of interview participants (employees) of interviews of the third iteration (N=15).

10.2196/67752 Multimedia Appendix 7 Interview guide for interviews of the fourth iteration.

10.2196/67752 Multimedia Appendix 8 Sample of interview participants (experts) of interviews of the fourth iteration (N=17).

10.2196/67752 Multimedia Appendix 9 Results of classification of “Likeminded” platform, “7Mind” app, and “Coaching & Counseling” service of “Fuerstenberg Institut” based on the new employee mental health program taxonomy, and methods and results of the interrater analysis.

10.2196/67752 Multimedia Appendix 10 Positionality statement.

10.2196/67752 Checklist 1 The 22-item PRISMA-ScR (Preferred Reporting Items for Systematic Reviews and Meta-Analyses Extension for Scoping Reviews) checklist for the first iteration.

10.2196/67752 Checklist 2 The 22-item PRISMA-ScR (Preferred Reporting Items for Systematic Reviews and Meta-Analyses extension for Scoping Reviews) checklist for the second iteration.

10.2196/67752 Checklist 3 The 32-item COREQ (Consolidated Criteria for Reporting Qualitative Research) checklist for interviews of the third iteration.

10.2196/67752 Checklist 4 The 32-item COREQ (Consolidated Criteria for Reporting Qualitative Research) checklist for interviews of the fourth iteration.

10.2196/67752 Checklist 5 The 32-item COREQ (Consolidated Criteria for Reporting Qualitative Research) checklist for the focus group.

10.2196/67752 Checklist 6 The 35-item ACCORD (Accurate Consensus Reporting Document) checklist for the focus group.

## Abbreviations

ACCORD: Accurate Consensus Reporting Document
COREQ: Consolidated Criteria for Reporting Qualitative Research
DMHI: digital mental health intervention
DMHT: digital mental health technology
EMHP: employee mental health program
*ICD-11*: *International Classification of Diseases, Eleventh Revision*
KOL: key opinion leader
PRISMA-ScR: Preferred Reporting Items for Systematic Reviews and Meta-Analyses Extension for Scoping Reviews

## References

[1] Patel V, Saxena S, Lund C (2023). Transforming mental health systems globally: principles and policy recommendations. Lancet.

[2] de Oliveira C, Saka M, Bone L, Jacobs R (2023). The role of mental health on workplace productivity: a critical review of the literature. Appl Health Econ Health Policy.

[3] Attridge M (2019). A global perspective on promoting workplace mental health and the role of employee assistance programs. Am J Health Promot.

[4] Strudwick J, Gayed A, Deady M (2023). Workplace mental health screening: a systematic review and meta-analysis. Occup Environ Med.

[5] Kelloway EK, Dimoff JK, Gilbert S (2023). Mental health in the workplace. Annu Rev Organ Psychol Organ Behav.

[6] Raggi A, Bernard RM, Toppo C (2024). The EMPOWER occupational e-mental health intervention implementation checklist to foster e-mental health interventions in the workplace: development study. J Med Internet Res.

[7] Sevov B, Huettemann R, Zinner M, Meister S, Fehring L (2025). Employee preference and use of employee mental health programs: mixed methods study. JMIR Hum Factors.

[8] Jeannotte AM, Hutchinson DM, Kellerman GR (2021). The time to change for mental health and wellbeing via virtual professional coaching: longitudinal observational study. J Med Internet Res.

[9] Joseph B, Walker A, Fuller-Tyszkiewicz M (2018). Evaluating the effectiveness of employee assistance programmes: a systematic review. Eur J Work Organ Psychol.

[10] Burger F, Neerincx MA, Brinkman WP (2020). Technological state of the art of electronic mental health interventions for major depressive disorder: systematic literature review. J Med Internet Res.

[11] Thomas BJ, Follmer KB, Meglich P (2024). Giving organization stakeholders better help: a taxonomy for making sense of workplace mental health offerings. Group Organ Manag.

[12] Armaou M, Konstantinidis S, Blake H (2020). The effectiveness of digital interventions for psychological well-being in the workplace: a systematic review protocol. Int J Environ Res Public Health.

[13] Economides M, Bolton H, Male R, Cavanagh K (2022). Feasibility and preliminary efficacy of web-based and mobile interventions for common mental health problems in working adults: multi-arm randomized pilot trial. JMIR Form Res.

[14] Roche A, Kostadinov V, Cameron J, Pidd K, McEntee A, Duraisingam V (2018). The development and characteristics of Employee Assistance Programs around the globe. J Workplace Behav Health.

[15] Attridge M (2023). The current state of employee assistance programs in the United States: a research-based commentary. Int J Sci Res Publ.

[16] Krisher L, Boeldt DL, Sigmon CAN, Rimel SE, Newman LS (2024). Pragmatic approach to the assessment and use of digital mental health interventions for health workers. Am J Public Health.

[17] Axén I, Björk Brämberg E, Vaez M, Lundin A, Bergström G (2020). Interventions for common mental disorders in the occupational health service: a systematic review with a narrative synthesis. Int Arch Occup Environ Health.

[18] Bouzikos S, Afsharian A, Dollard M, Brecht O (2022). Contextualising the effectiveness of an employee assistance program intervention on psychological health: the role of corporate climate. Int J Environ Res Public Health.

[19] Smail-Crevier R, Powers G, Noel C, Wang J (2019). Health-related internet usage and design feature preference for e-mental health programs among men and women. J Med Internet Res.

[20] Carolan S, Harris PR, Cavanagh K (2017). Improving employee well-being and effectiveness: systematic review and meta-analysis of web-based psychological interventions delivered in the workplace. J Med Internet Res.

[21] Phillips EA, Gordeev VS, Schreyögg J (2019). Effectiveness of occupational e-mental health interventions: a systematic review and meta-analysis of randomized controlled trials. Scand J Work Environ Health.

[22] Muñoz RF, Chavira DA, Himle JA (2018). Digital apothecaries: a vision for making health care interventions accessible worldwide. Mhealth.

[23] Liverpool S, Mota CP, Sales CMD (2020). Engaging children and young people in digital mental health interventions: systematic review of modes of delivery, facilitators, and barriers. J Med Internet Res.

[24] Gagnon MP, Sasseville M, Leblanc A (2022). Classification of digital mental health interventions: a rapid review and framework proposal. Stud Health Technol Inform.

[25] Pineda BS, Mejia R, Qin Y, Martinez J, Delgadillo LG, Muñoz RF (2023). Updated taxonomy of digital mental health interventions: a conceptual framework. Mhealth.

[26] Lukka L, Palva JM (2023). The development of game-based digital mental health interventions: bridging the paradigms of health care and entertainment. JMIR Serious Games.

[27] Ferrari M, Sabetti J, McIlwaine SV (2022). Gaming my way to recovery: a systematic scoping review of digital game interventions for young people’s mental health treatment and promotion. Front Digit Health.

[28] Bradley G, Rehackova L, Devereaux K (2025). Classifying the features of digital mental health interventions to inform the development of a patient decision aid. PLOS Digit Health.

[29] Hopkin G, Coole H, Edelmann F (2025). Toward a new conceptual framework for digital mental health technologies: scoping review. JMIR Ment Health.

[30] David E, DePierro JM, Marin DB, Sharma V, Charney DS, Katz CL (2022). COVID-19 pandemic support programs for healthcare workers and implications for occupational mental health: a narrative review. Psychiatr Q.

[31] LaMontagne AD, Martin A, Page KM (2014). Workplace mental health: developing an integrated intervention approach. BMC Psychiatry.

[32] Nickerson RC, Varshney U, Muntermann J (2013). A method for taxonomy development and its application in information systems. Eur J Inf Syst.

[33] Kundisch D, Muntermann J, Oberländer AM (2022). An update for taxonomy designers. Bus Inf Syst Eng.

[34] Tricco AC, Lillie E, Zarin W (2018). PRISMA extension for Scoping Reviews (PRISMA-ScR): checklist and explanation. Ann Intern Med.

[35] Braun V, Clarke V (2006). Using thematic analysis in psychology. Qual Res Psychol.

[36] Tong A, Sainsbury P, Craig J (2007). Consolidated criteria for reporting qualitative research (COREQ): a 32-item checklist for interviews and focus groups. Int J Qual Health Care.

[37] Szopinski D, Schoormann T, Kundisch D (2019). Because your taxonomy is worth it: towards a framework for taxonomy evaluation. https://aisel.aisnet.org/ecis2019_rp/104/.

[38] Fleiss JL (1971). Measuring nominal scale agreement among many raters. Psychol Bull.

[39] Gisev N, Bell JS, Chen TF (2013). Interrater agreement and interrater reliability: key concepts, approaches, and applications. Res Social Adm Pharm.

[40] Cohen J (1960). A coefficient of agreement for nominal scales. Educ Psychol Meas.

[41] Gattrell WT, Logullo P, van Zuuren EJ (2024). ACCORD (Accurate Consensus Reporting Document): a reporting guideline for consensus methods in biomedicine developed via a modified Delphi. PLOS Med.

[42] Meyermann A, Porzelt M (2014). Forschungsdatenzentrum (FDZ) Bildung Am DIPF (Deutsches Institut Für Internationale Pädagogische Forschung) [Guidelines for Anonymizing Qualitative Data].

[43] Johnson A, Gibson A (2014). Sustainability in Engineering Design.

[44] Möller F, Stachon M, Azkan C, Schoormann T, Otto B (2022). Designing business model taxonomies—synthesis and guidance from information systems research. Electron Markets.

[45] Scheutzow J, Attoe C, Harwood J (2022). Acceptability of web-based mental health interventions in the workplace: systematic review. JMIR Ment Health.

[46] International classification of diseases 11th revision (ICD-11). World Health Organization.

[47] Carolan S, de Visser RO (2018). Employees’ perspectives on the facilitators and barriers to engaging with digital mental health interventions in the workplace: qualitative study. JMIR Ment Health.

[48] Wan Mohd Yunus WMA, Musiat P, Brown JSL (2018). Systematic review of universal and targeted workplace interventions for depression. Occup Environ Med.

[49] Szeto ACH, Dobson KS (2010). Reducing the stigma of mental disorders at work: a review of current workplace anti-stigma intervention programs. Appl Prev Psychol.

[50] Dewa CS, Hoch JS (2015). Barriers to mental health service use among workers with depression and work productivity. J Occup Environ Med.

[51] Graham M, Milanowski A, Miller J (2012). Measuring and promoting inter-rater agreement of teacher and principal performance ratings. https://files.eric.ed.gov/fulltext/ED532068.pdf.

[52] Landis JR, Koch GG (1977). The measurement of observer agreement for categorical data. Biometrics.

[53] Denecke K, May R (2023). Developing a technical-oriented taxonomy to define archetypes of conversational agents in health care: literature review and cluster analysis. J Med Internet Res.

[54] Scheider S, Lauf F, Geller S, Möller F, Otto B (2023). Exploring design elements of personal data markets. Electron Markets.

[55] Weking J, Mandalenakis M, Hein A, Hermes S, Böhm M, Krcmar H (2020). The impact of blockchain technology on business models—a taxonomy and archetypal patterns. Electron Markets.

[56] Clay J, Eaton J, Gronholm PC, Semrau M, Votruba N (2020). Core components of mental health stigma reduction interventions in low- and middle-income countries: a systematic review. Epidemiol Psychiatr Sci.

[57] Chan S, Torous J, Hinton L, Yellowlees P (2015). Towards a framework for evaluating mobile mental health apps. Telemed J E Health.

[58] Nunes AP, Richmond MK, Pampel FC, Wood RC (2018). The effect of employee assistance services on reductions in employee absenteeism. J Bus Psychol.

[59] Baessler F, Zafar A, Gargot T (2021). Psychiatry training in 42 European countries: a comparative analysis. Eur Neuropsychopharmacol.

[60] Asian Federation of Psychiatric Associations (2016). The bulletin of the AFPA: the Summer 2016 Issue. Bull AFPA.

[61] Garabiles MR, Harper Shehadeh M, Hall BJ (2019). Cultural adaptation of a scalable World Health Organization e-mental health program for overseas Filipino workers. JMIR Form Res.

[62] Chowdhary N, Jotheeswaran AT, Nadkarni A (2014). The methods and outcomes of cultural adaptations of psychological treatments for depressive disorders: a systematic review. Psychol Med.

[63] Sit HF, Ling R, Lam AIF, Chen W, Latkin CA, Hall BJ (2020). The cultural adaptation of step-by-step: an intervention to address depression among Chinese young adults. Front Psychiatry.

[64] Cross S, Bell I, Nicholas J (2024). Use of AI in mental health care: community and mental health professionals survey. JMIR Ment Health.

[65] Olawade DB, Wada OZ, Odetayo A, David-Olawade AC, Asaolu F, Eberhardt J (2024). Enhancing mental health with artificial intelligence: current trends and future prospects. J Med Surg Public Health.

