# Supplementary material for: A Proposed Taxonomy to Holistically Classify Employee Mental Health Programs: Qualitative Taxonomy Development Study
Source: Interact J Med Res. 2025 Dec 18;14:e67752. doi: 10.2196/67752 (PMC12746229; doi:10.2196/67752)

**Multimedia Appendix 1. Screening process and final set of literature records of the first iteration.**


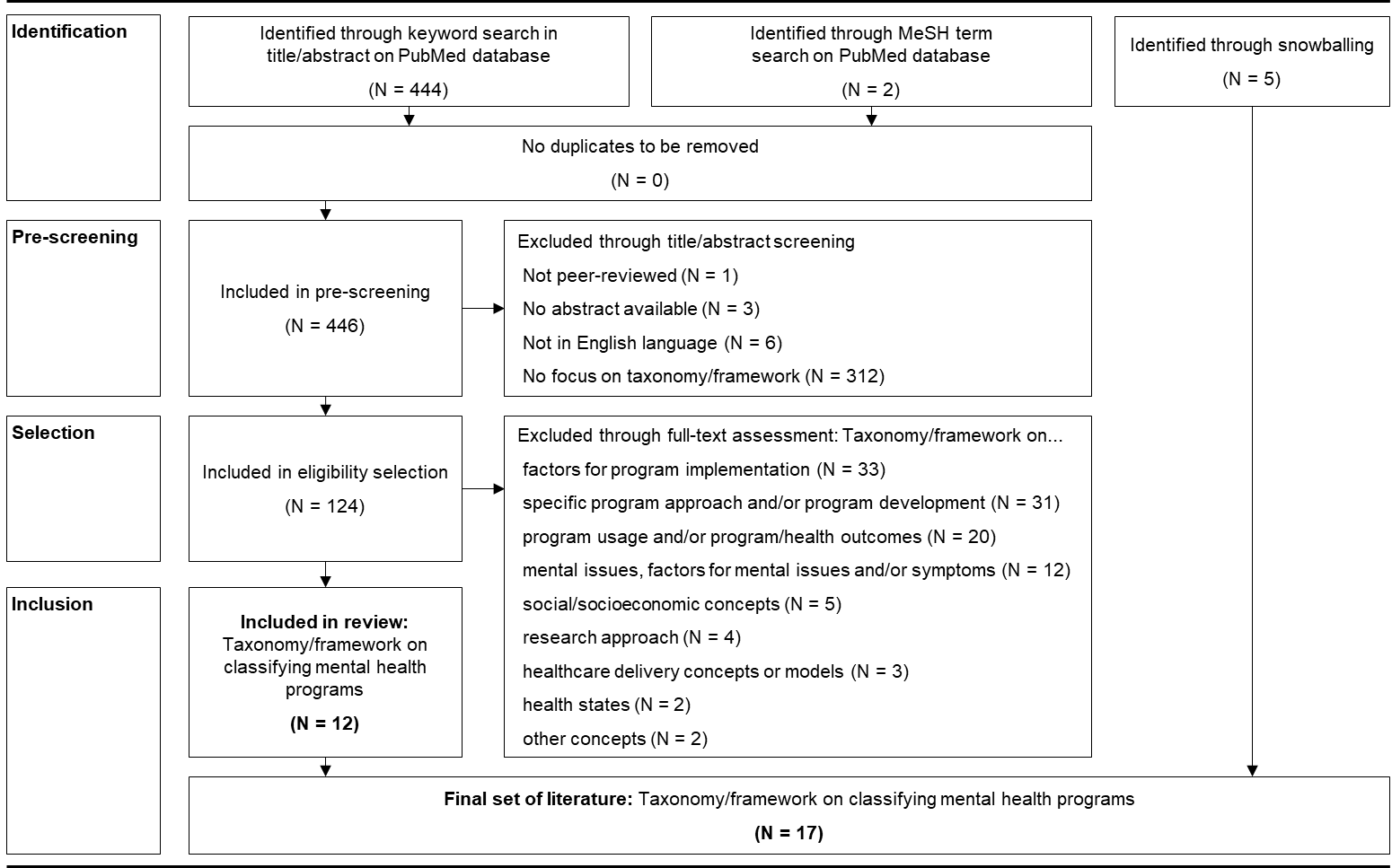

Supplement: Multimedia Appendix 1 [file ijmr-v14-e67752-s001.docx]
