# Supplementary material for: A Proposed Taxonomy to Holistically Classify Employee Mental Health Programs: Qualitative Taxonomy Development Study
Source: Interact J Med Res. 2025 Dec 18;14:e67752. doi: 10.2196/67752 (PMC12746229; doi:10.2196/67752)
Supplement: Multimedia Appendix 2 [file ijmr-v14-e67752-s002.docx]

**Multimedia Appendix 2. Overview of final set of literature records of the first iteration.**

| **Item** | **Title** | **DOI** | **Authors** | **Journal** | **Year of publication** | **Research focus** | **Derived codes** |
| --- | --- | --- | --- | --- | --- | --- | --- |
| *Scoping review* | | | | | | | |
| 1 | Technological State of the Art of Electronic Mental Health Interventions for Major Depressive Disorder: Systematic Literature Review | 10.2196/12599 | Burger et al. | Journal of Medical Internet Research | 2020 | Taxonomy of software systems for depression for DMHIs | D_1_ (C_1.1_, C_1.2_, C_1.3_); D_3_ (C_3.1_, C_3.2_, C_3.4_); D_6_ (C_6.1_); D_18_ (C_18.2_) |
| 2 | Core components of mental health stigma reduction interventions in low- and middle-income countries: a systematic review | 10.1017/S2045796020000797 | Clay et al. | Epidemiology and Psychiatric Sciences | 2020 | Framework on components of anti-stigma interventions | D_1_ (C_1.1_); D_3_ (C_3.1_); D_6_ (C_6.1_, C_6.2_); D_7_ (C_7.2_, C_7.3_); D_9_ (C_9.1_, C_9.3_); D_12_ (C_12.1_); D_16_ (C_16.1_, C_16.2_); D_18_ (C_18.2_) |
| 3 | COVID-19 Pandemic Support Programs for Healthcare Workers and Implications for Occupational Mental Health: A Narrative Review | 10.1007/s00420-020-01535-4 | David et al. | The Psychiatric Quarterly | 2022 | Basing/structuring paper fully on framework about integrated  approach to occupational mental health from LaMontagne et al. 2014 | D_1_ (C_1.1_, C_1.2_, C_1.3_) |
| 4 | Psychotherapy of adjustment disorders: Current state and future directions | 10.1080/15622975.2018.1467041 | Domhardt and Baumeister | The World Journal of Biological Psychiatry | 2018 | Stepped care approach framework for interventions | D_1_ (C_1.1_, C_1.2_, C_1.3_); D_3_ (C_3.1_, C_3.2_, C_3.3_); D_6_ (C_6.1_) |
| 5 | Gaming My Way to Recovery: A Systematic Scoping Review of Digital Game Interventions for Young People's Mental Health Treatment and Promotion | 10.3389/fdgth.2022.814248 | Ferrari et al. | Frontiers in Digital Health | 2022 | Stepped care approach framework for interventions and game elements of mental health interventions | D_1_ (C_1.1_, C_1.2_, C_1.3_); D_6_ (C_6.1_); D_11_ (C_11.3_) |
| 6 | Classification of Digital Mental Health Interventions: A Rapid Review and Framework Proposal | 10.3233/SHTI220545 | Gagnon et al. | Studies in Health Technology and Informatics | 2022 | Framework on digital mental health interventions | D_3_ (C_3.1_, C_3.2_, C_3.4_); D_6_ (C_6.1_); D_7_ (C_7.1_, C_7.2_, C_7.3_); D_8_ (C_8.1_, C_8.2_, C_8.3_); D_11_ (C_11.2_, C_11.3_); D_13_ (C_13.1_, C_13.2_) |
| 7 | Use of Technology to Promote Child Behavioral Health in the Context of Pediatric Care: A Scoping Review and Applications to Low- and Middle-Income Countries | 10.3389/fpsyt.2019.00806 | Huang et al. | Frontiers in Psychiatry | 2019 | Framework on electronic mental health interventions for children | D_16_ (C_16.1_, C_16.2_) |
| 8 | The Development of Game-Based Digital Mental Health Interventions: Bridging the Paradigms of Health Care and Entertainment | 10.2196/42173 | Lukka and Palva | JMIR Serious Games | 2023 | Intervention framework based on gamification degree | D_1_; D_3_; D_11_ (C_11.1_, C_11.2_, C_11.3_); D_14_; D_18_ (C_18.2_) |
| 9 | Updated taxonomy of digital mental health interventions: a conceptual framework | 10.21037/mhealth-23-6 | Pineda et al. | mHealth | 2023 | Framework on digital mental health interventions | D_1_ (C_1.1_, C_1.2_); D_2_ (C_2.1_, C_2.2_, C_2.3_, C_2.4_, C_2.6_); D_3_ (C_3.1_, C_3.4_); D_8_ (C_8.1_, C_8.2_, C_8.3_); D_9_ (C_9.1_, C_9.3_, C_9.4_); D_13_ (C_13.1_, C_13.2_); D_15_ (C_15.1_, C_15.2_) |
| 10 | The psychological impact of quarantine due to COVID-19: A systematic review of risk, protective factors and interventions using socio-ecological model framework | 10.1016/j.heliyon.2022.e09765 | Rajkumar et al. | Heliyon | 2022 | Framework on risk and protective factors and interventions for quarantine consequences based on Socio-Ecological Model (SEM) framework | D_16_ (C_16.1_, C_16.2_) |
| 11 | Developing a culturally and ecologically sound intervention program for youth exposed to war and terrorism | 10.1016/s1056-4993(02)00099-8 | Saltzman et al. | Child and Adolescent Psychiatric Clinics of North America | 2003 | 3 tiers framework on school MH interventions | D_1_ (C_1.1_, C_1.2_); D_3_ (C_3.2_); D_6_ (C_6.2_); D_7_ (C_7.2_, C_7.3_); D_9_ (C_9.1_); D_14_ (C_14.3_); D_15_ (C_15.1_, C_15.2_); D_18_ |
| 12 | Technology-Based Mental Health Interventions for Domestic Violence Victims Amid COVID-19 | 10.3390/ijerph19074286 | Su et al. | International Journal of Environmental Research and Public Health | 2022 | Socio-Ecological Model (SEM) framework | D_16_ (C_16.1_, C_16.2_) |
| *Snowballing* | | | | | | | |
| 1 | Towards a Framework for Evaluating Mobile Mental Health Apps | 10.1089/tmj.2015.0002 | Chan et al. | Telemedicine Journal and e-Health | 2015 | Framework on criteria to evaluate mental health apps | D_1_ (C_1.2_) |
| 2 | Workplace mental health: developing an integrated intervention approach | 10.1186/1471-244X-14-131 | LaMontagne et al. | BMC Psychiatry | 2014 | Three threads of integrated approach to workplace mental health | D_1_ (C_1.1_, C_1.2_, C_1.3_) |
| 3 | Engaging Children and Young People in Digital Mental Health Interventions: Systematic Review of Modes of Delivery, Facilitators, and Barriers | 10.2196/16317 | Liverpool et al. | Journal of Medical Internet Research | 2020 | Overview of modes of delivery of digital mental health interventions | D_6_ (C_6.1_) |
| 4 | Digital apothecaries: a vision for making health care interventions accessible worldwide | 10.21037/mhealth.2018.05.04 | Muñoz et al. | mHealth | 2018 | Taxonomy of types of face-to-face and digital interventions | D_6_ (C_6.1_, C_6.2_, C_6.3_); D_7_ (C_7.1_, C_7.2_); D_8_ (C_8.1_, C_8.2_, C_8.3_); D_13_ (C_13.1_, C_13.2_) |
| 5 | Fostering Healthy Mental, Emotional, and Behavioral Development in Children and Youth: A National Agenda | 10.17226/25201 | National Academies Press (US) | National Academies Press (US) | 2019 | Framework on fostering mental health | D_1_ (C_1.1_, C_1.2_, C_1.3_) |

**References:**

1. Burger F, Neerincx MA, Brinkman WP. Technological state of the art of electronic mental health interventions for major depressive disorder: systematic literature review. J Med Internet Res. Jan 20, 2020;22(1):e12599. doi: 10.2196/12599. Medline: 31958063.
2. Clay J, Eaton J, Gronholm PC, Semrau M, Votruba N. Core components of mental health stigma reduction interventions in low- and middle-income countries: a systematic review. Epidemiol Psychiatr Sci. Sep 4, 2020;29:e164. doi: 10.1017/S2045796020000797. Medline: 32883399.
3. David E, DePierro JM, Marin DB, Sharma V, Charney DS, Katz CL. COVID-19 pandemic support programs for healthcare workers and implications for occupational mental health: a narrative review. Psychiatr Q. Mar 2022;93(1):227-247. doi: 10.1007/s11126-021-09952-5. Medline: 34606067.
4. Domhardt M, Baumeister H. Psychotherapy of adjustment disorders: current state and future directions. World J Biol Psychiatry. 2018;19(sup1):S21-S35. doi: 10.1080/15622975.2018.1467041. Medline: 30204563.
5. Ferrari M, Sabetti J, McIlwaine SV, et al. Gaming my way to recovery: a systematic scoping review of digital game interventions for young people’s mental health treatment and promotion. Front Digit Health. 2022;4:814248. doi: 10.3389/fdgth.2022.814248. Medline: 35465647.
6. Gagnon MP, Sasseville M, Leblanc A. Classification of digital mental health interventions: a rapid review and framework proposal. Stud Health Technol Inform. May 25, 2022;294:629-633. doi: 10.3233/SHTI220545. Medline: 35612165.
7. Huang KY, Lee D, Nakigudde J, Cheng S, et al. Use of technology to promote child behavioral health in the context of pediatric care: a scoping review and applications to low- and middle-income countries. Front Psychiatry. Nov 13, 2019;10:806. doi: 10.3389/fpsyt.2019.00806. Medline: 31798470.
8. Lukka L, Palva JM. The development of game-based digital mental health interventions: bridging the paradigms of health care and entertainment. JMIR Serious Games. Sep 4, 2023;11:e42173. doi: 10.2196/42173. Medline: 37665624.
9. Pineda BS, Mejia R, Qin Y, Martinez J, Delgadillo LG, Muñoz RF. Updated taxonomy of digital mental health interventions: a conceptual framework. Mhealth. 2023;9(28):1-17. doi: 10.21037/mhealth-23-6. Medline: 37492117.
10. Rajkumar E, Rajan AM, Daniel M, et al. The psychological impact of quarantine due to COVID-19: a systematic review of risk, protective factors and interventions using socio-ecological model framework. Heliyon. Jun 2022;8(6):e09765. doi: 10.1016/j.heliyon.2022.e09765. Medline: 35756104.
11. Saltzman WR, Layne CM, Steinberg AM, Arslanagic B, Pynoos RS. Developing a culturally and ecologically sound intervention program for youth exposed to war and terrorism. Child Adolesc Psychiatr Clin N Am. Apr 2003;12(2):319-342. doi: 10.1016/s1056-4993(02)00099-8. Medline: 12725014.
12. Su Z, Cheshmehzangi A, McDonnell D, et al. Technology-based mental health interventions for domestic violence victims amid COVID-19. Int J Environ Res Public Health. Apr 3, 2022;19(7):4286. doi: 10.3390/ijerph19074286. Medline: 35409967.
13. Chan S, Torous J, Hinton L, Yellowlees P. Towards a framework for evaluating mobile mental health apps. Telemed J E Health. Dec 2015;21(12):1038-1041. doi: 10.1089/tmj.2015.0002. Medline: 26171663.
14. LaMontagne AD, Martin A, Page KM, et al. Workplace mental health: developing an integrated intervention approach. BMC Psychiatry. May 9, 2014;14:131. doi: 10.1186/1471-244X-14-131. Medline: 24884425.
15. Liverpool S, Mota CP, Sales CMD, et al. Engaging children and young people in digital mental health interventions: systematic review of modes of delivery, facilitators, and barriers. J Med Internet Res. 2020;22(6):e16317. doi: 10.2196/ 16317. Medline: 32442160.
16. Muñoz RF, Chavira DA, Himle JA, et al. Digital apothecaries: a vision for making health care interventions accessible worldwide. Mhealth. 2018;4:18. doi: 10.21037/mhealth.2018.05.04. Medline: 30050914.
17. National Academies of Sciences, Engineering, and Medicine; Division of Behavioral and Social Sciences and Education; Board on Children, Youth, and Families; Committee on Fostering Healthy Mental, Emotional, and Behavioral Development Among Children and Youth. Fostering healthy mental, emotional, and behavioral development in children and youth: a national agenda. National Academies Press (US); Sep 11, 2019. doi: 10.17226/25201. Medline: 31869055.
