# Supplementary material for: A Proposed Taxonomy to Holistically Classify Employee Mental Health Programs: Qualitative Taxonomy Development Study
Source: Interact J Med Res. 2025 Dec 18;14:e67752. doi: 10.2196/67752 (PMC12746229; doi:10.2196/67752)
Supplement: Multimedia Appendix 3 [file ijmr-v14-e67752-s003.docx]

**Multimedia Appendix 3.** Screening process and final set of literature records of the second iteration.


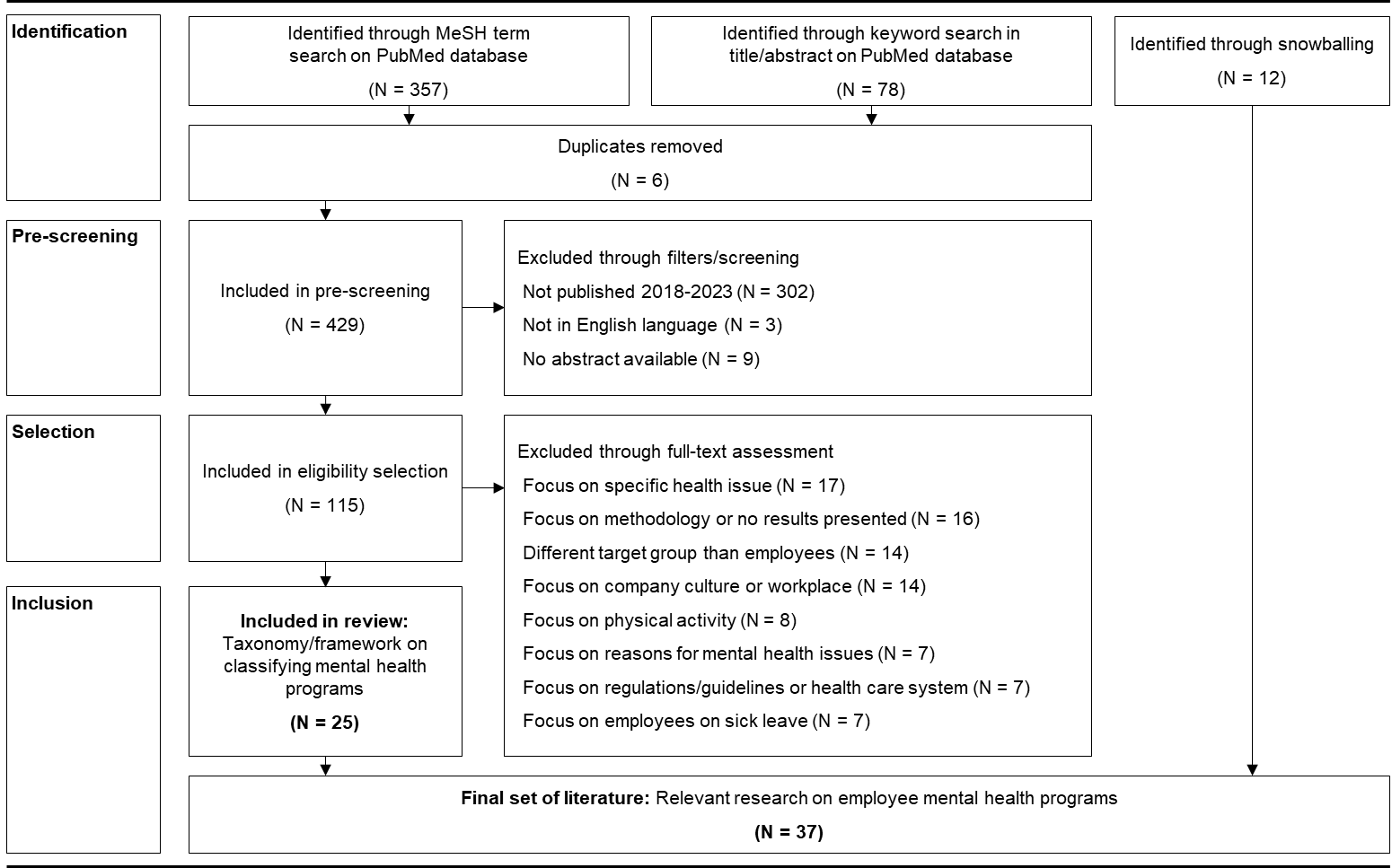


Note: This scoping review was conducted in the context of a larger research project on EMHPs. Thus, the screening process and the final set of literature records were similar to that of another study by Sevov et al., as is this visualization of the screening process, but the identified literature records were analyzed differently per study as both studies had distinct research objectives. The overview of the scoping review process of the other study can be found in its Figure 1 (originally published in JMIR Human Factors under the terms of the [Creative Commons Attribution License 4.0](https://creativecommons.org/licenses/by/4.0/), <https://humanfactors.jmir.org/2025/1/e65750/>; Sevov B, Huettemann R, Zinner M, Meister S, Fehring L; Employee Preference and Use of Employee Mental Health Programs: Mixed Methods Study; JMIR Hum Factors 2025; 12:e65750; doi: [10.2196/65750](https://doi.org/10.2196/65750)).
