# Supplementary material for: A Proposed Taxonomy to Holistically Classify Employee Mental Health Programs: Qualitative Taxonomy Development Study
Source: Interact J Med Res. 2025 Dec 18;14:e67752. doi: 10.2196/67752 (PMC12746229; doi:10.2196/67752)
Supplement: Multimedia Appendix 4 [file ijmr-v14-e67752-s004.docx]

**Multimedia Appendix 4. Overview of final set of literature records of the second iteration.**

| **Item** | **Title** | **DOI** | **Authors** | **Journal** | **Year of publication** | **Research focus** | **Derived codes** |
| --- | --- | --- | --- | --- | --- | --- | --- |
| *Scoping review* | | | | | | | |
| 1 | The Effectiveness of Digital Interventions for Psychological Well-Being in the Workplace: A Systematic Review Protocol | 10.3390/ijerph17010255 | Armaou et al. | International Journal of Environmental Research and Public Health | 2020 | Effectiveness of EMHPs | (C_1.1_, C_1.2_); (C_2.1_, C_2.2_, C_2.3_); (C_3.1_, C_3.4_); D_6_ (C_6.1_); (C_7.2_, C_7.3_); D_13_; (C_18.1_) |
| 2 | A Global Perspective on Promoting Workplace Mental Health and the Role of Employee Assistance Programs | 10.1177/0890117119838101c | Attridge | American Journal of Health Promotion | 2019 | Meaning of EMHPs on promoting mental health at the workplace | (C_1.2_); (C_3.4_); (C_17.1_, C_17.2_); D_20_ |
| 3 | Interventions for common mental disorders in the occupational health service: a systematic review with a narrative synthesis | 10.1007/s00420-020-01535-4 | Axén et al. | International Archives of Occupational and Environmental Health | 2020 | Effectiveness of EMHPs | (C_1.1_, C_1.2_, C_1.3_); (C_2.4_); (C_3.1_, C_3.3_, C_3.4_); (C_6.1_); (C_7.1_, C_7.2_, C_7.3_); (C_9.1_); (C_14.2_) |
| 4 | Professional training in mental health self-care for nurses starting work in hospital departments | 10.3233/WOR-203311 | Bernburg et al. | Work | 2020 | Effectiveness of EMHPs on healthcare workers | (C_3.1_, C_3.4_); (C_7.3_); (C_9.1_); (C_18.2_) |
| 5 | Contextualising the Effectiveness of an Employee Assistance Program Intervention on Psychological Health: The Role of Corporate Climate | 10.3390/ijerph19095067 | Bouzikos et al. | International Journal of Environmental Research and Public Health | 2022 | Effectiveness of EMHPs | (C_1.1_); D_2_ (C_2.1_, C_2.2_, C_2.3_, C_2.6_, C_2.7_); D_3_ (C_3.4_); (C_6.1_, C_6.2_); (C_7.2_); D_9_ (C_9.1_); (C_16.1_, C_16.2_) |
| 6 | Economic analyses of mental health and substance use interventions in the workplace: a systematic literature review and narrative synthesis | 10.1016/S2215-0366(20)30145-0 | de Oliveira et al. | The Lancet Psychiatry | 2020 | Economic analyses of EMHPs | (C_1.1_, C_1.2_); D_2_ (C_2.1_, C_2.2_, C_2.3_, C_2.6_, C_2.7_); (C_3.1_, C_3.3_, C_3.4_); (C_6.1_); (C_7.2_); (C_8.3_); (C_9.1_) |
| 7 | With a little help from my boss: The impact of workplace mental health training on leader behaviors and employee resource utilization | 10.1037/ocp0000126 | Dimoff and Kelloway | Journal of Occupational Health Psychology | 2019 | Impact of leadership on employees' usage of EMHPs | (C_1.1_); D_2_ (C_2.1_, C_2.2_, C_2.3_, C_2.6_); (C_6.1_, C_6.2_); (C_7.2_, C_7.3_); (C_8.1_, C_8.2_); (C_10.1_); (C_14.1_); (C_17.2_); (C_20.1_) |
| 8 | Effects of the Anti-stigma Workplace Intervention "Working Mind" in a Canadian Health-Care Setting: A Cluster-Randomized Trial of Immediate Versus Delayed Implementation | 10.1177/0706743720961738 | Dobson et al. | Canadian Journal of Psychiatry | 2021 | Effectiveness of EMHPs | (C_7.2_, C_7.3_); D_18_ |
| 9 | Workplace Support for Mental Health Workers Who Are Parents: A Feasibility Study | 10.3389/fpsyg.2022.8540652 | Dunn et al. | Frontiers in Psychology | 2022 | Effectiveness of EMHPs | (C_3.1_) |
| 10 | Psychotherapeutic Consultation Services in the Workplace: A Longitudinal Analysis of Treatments and Sick Leave Using Health Insurance Data | 10.3389/fpsyt.2022.838823 | Gantner et al. | Frontiers in Psychiatry | 2022 | Effectiveness of EMHPs; characteristics of users | (C_3.2_); (C_5.1_); D_7_ (C_7.1_); (C_8.2_); D_9_ (C_9.1_); D_14_ (C_14.2_); D_15_ (C_15.1_); D_18_; D_20_ (C_20.1_) |
| 11 | Mental Health in the Workplace: A Call to Action Proceedings From the Mental Health in the Workplace-Public Health Summit | 10.1097/JOM.0000000000001271 | Goetzel et al. | Journal of Occupational and Environmental Medicine | 2018 | Workplace health promotion through effective programs | (C_1.1_); (C_2.1_, C_2.6_, C_2.7_); D_3_ (C_3.1_, C_3.3_); (C_5.2_); D_6_ (C_6.1_, C_6.3_); D_16_ (C_16.1_); (C_20.1_, C_20.2_) |
| 12 | Workplace-Based Organizational Interventions Promoting Mental Health and Happiness among Healthcare Workers: A Realist Review | 10.3390/ijerph16224396 | Gray et al. | International Journal of Environmental Research and Public Health | 2019 | Effectiveness of EMHPs on healthcare workers | (C_1.1_); D_2_ (C_2.2_, C_2.3_, C_2.6_, C_2.7_); D_3_ (C_3.1_, C_3.4_); D_15_ (C_15.1_, C_15.2_); (C_16.1_); D_17_ (C_17.2_); D_18_ (C_18.2_) |
| 13 | Testing the Pragmatic Effectiveness of a Consumer-Based Mindfulness Mobile App in the Workplace: Randomized Controlled Trial | 10.2196/38903 | Huberty et al. | JMIR mHealth and uHealth | 2022 | Effectiveness of EMHPs | (C_2.1_, C_2.2_, C_2.3_, C_2.4_); (C_3.4_); (C_6.1_, C_6.2_); (C_7.2_, C_7.3_); D_13_ (C_13.1_); D_14_ (C_14.2_); (C_20.1_) |
| 14 | The Impact of Personalized Human Support on Engagement With Behavioral Intervention Technologies for Employee Mental Health: An Exploratory Retrospective Study | 10.3389/fdgth.2022.846375 | Jesuthasan et al. | Frontiers in Digital Health | 2022 | Factors for engagement with EMHPs | (C_3.1_, C_3.4_); (C_6.1_, C_6.2_); (C_7.1_, C_7.2_); D_8_ (C_8.1_, C_8.3_); D_9_ (C_9.1_); D_10_ (C_10.1_); (C_14.2_) |
| 15 | Effects of gratitude intervention on mental health and well-being among workers: A systematic review | 10.1002/1348-9585.12290 | Komase et al. | Journal of Occupational Health | 2021 | Effectiveness of gratitude EMHP | (C_2.1_, C_2.2_, C_2.3_); D_3_ (C_3.4_); (C_6.1_, C_6.2_); (C_7.3_); (C_10.1_); D_13_ (C_13.2_); D_14_ (C_14.2_); (C_15.1_); (C_18.2_) |
| 16 | A multi-faceted community intervention is associated with knowledge and standards of workplace mental health: the Superior Mental Wellness @ Work study | 10.1186/s12889-019-6976-x | Kristman et al. | BMC Public Health | 2019 | Program effectiveness on mental health awareness and knowledge | (C_3.1_, C_3.4_); (C_7.3_); (C_14.2_) |
| 17 | Efficacy of a Workplace Intervention Program With Web-Based Online and Offline Modalities for Improving Workers' Mental Health | 10.3389/fpsyt.2022.888157 | Lam et al. | Frontiers in Psychiatry | 2022 | Effectiveness of EMHPs | (C_1.1_); D_2_ (C_2.1_, C_2.2_, C_2.3_, C_2.7_); (C_6.1_, C_6.2_, C_6.3_); (C_7.1_, C_7.2_, C_7.3_); (C_8.1_, C_8.2_); (C_13.2_); (C_14.2_); D_16_ (C_16.1_) |
| 18 | Mental health promotion for junior physicians working in emergency medicine: evaluation of a pilot study | 10.1097/MEJ.0000000000000434 | Mache et al. | European Journal of Emergency Medicine | 2018 | Effectiveness of EMHPs on healthcare workers | (C_1.1_); (C_3.1_, C_3.4_); (C_6.1_, C_6.2_); (C_7.3_); (C_9.1_); (C_12.1_, C_12.2_); (C_14.2_) |
| 19 | Exploring men's use of mental health support offered by an Australian Employee Assistance Program (EAP): perspectives from a focus-group study with males working in blue- and white-collar industries | 10.1186/s13033-021-00489-5 | Matthews et al. | International Journal of Mental Health Systems | 2021 | Factors for EMHP usage for male employees | (C_1.1_); (C_3.4_); (C_6.1_); D_18_ (C_18.2_); (C_20.1_) |
| 20 | The effectiveness of workplace health promotion program in reducing work-related depression, anxiety and stress among manufacturing workers in Malaysia: mixed-model intervention | 10.1007/s00420-022-01836-w | Mohamed et al. | International Archives of Occupational and Environmental Health | 2022 | Effectiveness of EMHPs on manufacturing workers | (C_1.1_); (C_2.1_, C_2.2_, C_2.3_, C_2.4_); (C_3.1_, C_3.4_); (C_7.2_, C_7.3_); (C_10.1_); (C_14.2_); D_16_ (C_16.1_); D_18_ (C_18.2_) |
| 21 | Effects of a work-related stress model based mental health promotion program on job stress, stress reactions and coping profiles of women workers: a control groups study | 10.1186/s12889-020-09769-0 | Ornek and Esin | BMC Public Health | 2020 | Effectiveness of EMHPs on female workers | (C_1.1_); D_2_ (C_2.3_, C_2.7_); (C_3.4_); (C_6.1_, C_6.2_); (C_7.3_); (C_8.3_); (C_12.1_); D_13_ (C_13.1_); (C_18.2_) |
| 22 | Workplace Mental Health Interventions in India: A Rapid Systematic Scoping Review | 10.3389/fpubh.2022.800880 | Pandya et al. | Frontiers in Public Health | 2022 | EMHP landscape in India | (C_1.1_); D_2_ (C_2.3_); (C_6.1_); (C_7.2_, C_7.3_); (C_10.1_); (C_19.1_, C_19.2_) |
| 23 | Beyond the Lab: Empirically Supported Treatments in the Real World | 10.3389/fpsyg.2020.01969 | Schneider et al. | Frontiers in Psychology | 2020 | Effectiveness of EMHPs | D_2_ (C_2.1_, C_2.2_); (C_3.1_, C_3.2_, C_3.3_); (C_7.2_, C_7.3_); (C_8.3_); (C_9.1_); (C_14.2_); (C_16.1_) |
| 24 | Systematic review of universal and targeted workplace interventions for depression | 10.1136/oemed-2017-104532 | Wan Mohd Yunus et al. | Occupational and Environmental Medicine | 2018 | Landscape and effectiveness of EMHPs | (C_1.1_, C_1.2_); (C_2.1_, C_2.2_, C_2.6_, C_2.7_); D_3_ (C_3.1_, C_3.2_, C_3.3_, C_3.4_); D_6_ (C_6.1_, C_6.2_, C_6.3_); (C_7.1_, C_7.3_); (C_14.2_); D_16_ (C_16.1_, C_16.2_); (C_18.1_, C_18.2_) |
| 25 | Effect of EAP Psychological Intervention on Improving the Mental Health of Medical Workers Under the Novel Coronavirus Epidemic in China | 10.3389/fpubh.2021.6491572 | Xu et al. | Frontiers in Public Health | 2021 | Effectiveness of EMHPs on healthcare workers | (C_2.1_, C_2.2_, C_2.7_); (C_3.4_); (C_6.1_); (C_7.2_, C_7.3_); (C_8.3_); (C_9.1_); (C_10.1_); (C_14.2_, C_14.3_); (C_18.2_) |
| *Snowballing* | | | | | | | |
| 1 | Improving Employee Well-Being and Effectiveness: Systematic Review and Meta-Analysis of Web-Based Psychological Interventions Delivered in the Workplace | 10.2196/jmir.7583 | Carolan et al. | Journal of Medical Internet Research | 2017 | Effectiveness of EMHPs | (C_2.1_, C_2.3_); D_3_ (C_3.1_, C_3.4_); D_6_ (C_6.1_); (C_8.1_, C_8.2_); D_9_ (C_9.1_, C_9.3_); D_14_ (C_14.2_); (C_17.2_); D_18_ (C_18.2_) |
| 2 | Employees' Perspectives on the Facilitators and Barriers to Engaging With Digital Mental Health Interventions in the Workplace: Qualitative Study | 10.2196/mental.9146 | Carolan and de Visser | JMIR Mental Health | 2018 | Factors for EMHP usage | (C_3.1_); (C_6.1_, C_6.2_); (C_7.2_, C_7.3_); (C_8.1_, C_8.2_); (C_10.1_); (C_12.1_, C_12.2_, C_12.3_); (C_13.1_, C_13.2_); (C_14.2_); (C_15.2_, C_15.3_); D_18_ (C_18.2_) |
| 3 | Predictors of Psychological Distress and Mental Health Resource Utilization among Employees in Malaysia | 10.3390/ijerph18010314 | Chan et al. | International Journal of Environmental Research and Public Health | 2021 | Factors for EMHP usage | D_2_ (C_2.1_, C_2.2_, C_2.7_); (C_3.1_, C_3.2_, C_3.4_); (C_5.2_); (C_6.1_, C_6.2_) |
| 4 | Barriers to Mental Health Service Use Among Workers With Depression and Work Productivity | 10.1097/JOM.000000000000047 | Dewa and Hoch | Journal of Occupational and Environmental Medicine | 2015 | Factors for EMHP usage; economic effect of removing barriers | (C_3.4_); (C_20.2_) |
| 5 | Evaluating the effectiveness of employee assistance programmes: a systematic review | 10.1080/1359432X.2017.1374245 | Joseph et al. | European Journal of Work and Organizational Psychology | 2018 | Effectiveness of EMHPs | (C_1.2_); D_2_ (C_2.3_, C_2.6_); (C_3.4_); D_6_ (C_6.1_, C_6.2_); (C_7.3_); (C_8.3_); D_14_; (C_17.2_); D_18_ (C_18.1_, C_18.2_); D_19_ (C_19.1_, C_19.2_) |
| 6 | The Effect of Employee Assistance Services on Reductions in Employee Absenteeism | 10.1007/s10869-017-9518-5 | Nunes et al. | Journal of Business and Psychology | 2018 | Effectiveness of EMHPs | D_2_ (C_2.1_, C_2.2_, C_2.6_); D_3_ (C_3.1_, C_3.4_); (C_6.1_); (C_7.2_); (C_9.1_); (C_14.2_); (C_19.1_, C_19.2_); (C_20.1_) |
| 7 | Worker Preferences for a Mental Health App Within Male-Dominated Industries: Participatory Study | 10.2196/mental.8999 | Peters et al. | JMIR Mental Health | 2018 | Preference of employees for different types of mental health programs by gender | (C_1.1_); (C_3.1_, C_3.4_); (C_6.1_, C_6.2_); (C_7.1_, C_7.2_); (C_12.1_); (C_14.2_); (C_18.2_); D_19_ (C_19.1_, C_19.2_) |
| 8 | Effectiveness of occupational e-mental health interventions: a systematic review and meta-analysis of randomized controlled trials | 10.5271/sjweh.3839 | Phillips et al. | Scandinavian Journal of Work, Environment & Health | 2019 | Effectiveness of EMHPs | D_2_ (C_2.1_, C_2.2_, C_2.3_, C_2.4_, C_2.6_); D_3_ (C_3.1_, C_3.4_); D_6_ (C_6.1_, C_6.2_); (C_7.1_, C_7.2_, C_7.3_); D_8_ (C_8.1_, C_8.3_); (C_9.1_); (C_18.2_) |
| 9 | Acceptability of Web-Based Mental Health Interventions in the Workplace: Systematic Review | 10.2196/34655 | Scheutzow et al. | JMIR Mental Health | 2022 | Acceptability and usage factors of employees regarding EMHPs | (C_1.1_, C_1.2_, C_1.3_); D_2_ (C_2.1_, C_2.2_, C_2.3_, C_2.4_, C_2.6_, C_2.7_); D_3_ (C_3.1_, C_3.4_); (C_6.1_); (C_7.1_); D_8_ (C_8.1_); (C_10.1_); (C_12.1_); (C_14.2_); (C_15.1_); D_18_ (C_18.1_, C_18.2_) |
| 10 | Health-Related Internet Usage and Design Feature Preference for E-Mental Health Programs Among Men and Women | 10.2196/11224 | Smail-Crevier et al. | Journal of Medical Internet Research | 2019 | Preference for different types of EMHPs by gender | D_1_ (C_1.1_, C_1.2_); (C_2.1_, C_2.3_); (C_3.1_); D_6_ (C_6.1_, C_6.2_); (C_7.1_, C_7.2_); (C_8.1_, C_8.2_, C_8.3_); (C_9.1_); (C_10.1_); (C_12.2_) |
| 11 | Effectiveness of eHealth interventions for reducing mental health conditions in employees: A systematic review and meta-analysis | 10.1371/journal.pone.0189904 | Stratton et al. | PLOS One | 2017 | Preference for different types of EMHPs | (C_1.1_, C_1.2_); D_2_ (C_2.1_, C_2.2_, C_2.3_, C_2.6_); D_3_ (C_3.1_, C_3.4_); (C_6.1_); (C_8.1_, C_8.3_); (C_14.2_); (C_16.1_, C_16.2_); (C_18.1_); (C_19.1_, C_19.2_) |
| 12 | Support for depression in the workplace: Perspectives  of employees, managers, and OHS personnel | 10.1007/s41542-021-00090-9 | van Eerd et al. | Occupational Health Science | 2021 | Preference and factors for usage of mental health resources at the workplace incl. barriers | (C_1.1_); (C_2.1_, C_2.2_, C_2.3_); (C_3.1_, C_3.3_); D_6_ (C_6.1_, C_6.2_); (C_9.1_); (C_14.1_); (C_15.1_, C_15.2_, C_15.3_); (C_17.1_, C_17.2_); (C_19.2_) |

Note: This scoping review was conducted in the context of a larger research project on EMHPs. Thus, the identified literature records were also used and analyzed for another study by Sevov et al., but they were analyzed differently per study as both studies had distinct research objectives. Given the same literature records were used in both studies, this overview of the final set of literature records is similar to that of the other study, but the overviews differ in the derived insights they present. The overview of the identified literature of the other study can be found in its [Multimedia Appendix 6](https://jmir.org/api/download?alt_name=humanfactors_v12i1e65750_app6.docx&filename=e3e764c708537221b6a321017e5d0044.docx) (originally published in JMIR Human Factors under the terms of the [Creative Commons Attribution License 4.0](https://creativecommons.org/licenses/by/4.0/), <https://humanfactors.jmir.org/2025/1/e65750/>; Sevov B, Huettemann R, Zinner M, Meister S, Fehring L; Employee Preference and Use of Employee Mental Health Programs: Mixed Methods Study; JMIR Hum Factors 2025; 12:e65750; doi: [10.2196/65750](https://doi.org/10.2196/65750)).

**References:**

1. Armaou M, Konstantinidis S, Blake H. The effectiveness of digital interventions for psychological well-being in the workplace: a systematic review protocol. Int J Environ Res Public Health. 2020;17(1):255. doi: 10.3390/ijerph17010255. Medline: 31905882.
2. Attridge M. A global perspective on promoting workplace mental health and the role of employee assistance programs. Am J Health Promot. May 2019;33(4):622-629. doi: 10.1177/0890117119838101c. Medline: 31006254.
3. Axén I, Björk Brämberg E, Vaez M, Lundin A, Bergström G. Interventions for common mental disorders in the occupational health service: a systematic review with a narrative synthesis. Int Arch Occup Environ Health. Oct 2020;93(7):823-838. doi: 10.1007/s00420-020-01535-4. Medline: 32246230.
4. Bernburg M, Groneberg D, Mache S. Professional training in mental health self-care for nurses starting work in hospital departments. Work. 2020;67(3):583-590. doi: 10.3233/WOR-203311. Medline: 33185622.
5. Bouzikos S, Afsharian A, Dollard M, Brecht O. Contextualising the effectiveness of an employee assistance program intervention on psychological health: the role of corporate climate. Int J Environ Res Public Health. Apr 21, 2022;19(9):5067. doi: 10.3390/ijerph19095067. Medline: 35564466.
6. de Oliveira C, Cho E, Kavelaars R, Jamieson M, Bao B, Rehm J. Economic analyses of mental health and substance use interventions in the workplace: a systematic literature review and narrative synthesis. Lancet Psychiatry. Oct 2020;7(10):893-910. doi: 10.1016/S2215-0366(20)30145-0. Medline: 32949521.
7. Dimoff JK, Kelloway EK. With a little help from my boss: the impact of workplace mental health training on leader behaviors and employee resource utilization. J Occup Health Psychol. Feb 2019;24(1):4-19. doi: 10.1037/ocp0000126. Medline: 29939045.
8. Dobson KS, Markova V, Wen A, Smith LM. Effects of the anti-stigma workplace Intervention “Working Mind” in a Canadian health-care setting: a cluster-randomized trial of immediate versus delayed implementation. Can J Psychiatry. May 2021;66(5):495-502. doi: 10.1177/0706743720961738. Medline: 32960651.
9. Dunn A, Dixon C, Thomson A, Cartwright-Hatton S. Workplace support for mental health workers who are parents: a feasibility study. Front Psychol. Jun 23, 2022;13:854065. doi: 10.3389/fpsyg.2022.8540652. Medline: 35814147.
10. Gantner M, Jarzcok MN, Schneider J, Brandner S, Gündel H, Wietersheim J von. Psychotherapeutic consultation services in the workplace: a longitudinal Analysis of treatments and sick leave using health insurance data. Front. Psychiatry. Mar 24, 2022;13:838823. doi: 10.3389/fpsyt.2022.838823. Medline: 35401269.
11. Goetzel RZ, Roemer EC, Holingue C, et al. Mental health in the workplace: a call to action proceedings from the mental health in the workplace-public health summit. J Occup Environ Med. Apr 2018;60(4):322-330. doi: 10.1097/JOM.0000000000001271. Medline: 29280775.
12. Gray P, Senabe S, Naicker N, Kgalamono S, Yassi A, Spiegel JM. Workplace-based organizational interventions promoting mental health and happiness among healthcare workers: a realist review. Int J Environ Res Public Health. Nov 11, 2019;16(22):4396. doi: 10.3390/ijerph16224396. Medline: 31717906.
13. Huberty JL, Espel-Huynh HM, Neher TL, Puzia ME. Testing the pragmatic effectiveness of a consumer-based mindfulness mobile app in the workplace: randomized controlled trial. JMIR Mhealth Uhealth. Sep 28, 2022;10(9):e38903. doi: 10.2196/38903. Medline: 36169991.
14. Jesuthasan J, Low M, Ong T. The impact of personalized human support on engagement with behavioral intervention technologies for employee mental health: an exploratory retrospective study. Front Digit Health. Apr 27, 2022;4:846375. doi: 10.3389/fdgth.2022.846375. Medline: 35574254.
15. Komase Y, Watanabe K, Hori D, et al. Effects of gratitude intervention on mental health and well-being among workers: a systematic review. J Occup Health. Jan 2021;63(1):e12290. doi: 10.1002/1348-9585.12290. Medline: 34762326.
16. Kristman VL, Lowey J, Fraser L, Armstrong S, Sawula S. A multi-faceted community intervention is associated with knowledge and standards of workplace mental health: the Superior Mental Wellness @ Work study. BMC Public Health. May 24, 2019;19(1):638. doi: 10.1186/s12889-019-6976-x. Medline: 31126273.
17. Lam LT, Lam MK, Reddy P, Wong P. Efficacy of a workplace intervention program with web-based online and offline modalities for improving workers’ mental health. Front. Psychiatry. May 31, 2022;13:888157. doi: 10.3389/fpsyt.2022.888157. Medline: 35711597.
18. Mache S, Bernburg M, Baresi L, Groneberg D. Mental health promotion for junior physicians working in emergency medicine: evaluation of a pilot study. Eur J Emerg Med. Jun 2018;25(3):191-198. doi: 10.1097/MEJ.0000000000000434. Medline: 27879536.
19. Matthews LR, Gerald J, Jessup GM. Exploring men’s use of mental health support offered by an Australian Employee Assistance Program (EAP): perspectives from a focus-group study with males working in blue- and white-collar industries. Int J Ment Health Syst. Aug 4, 2021;15:68. doi: 10.1186/s13033-021-00489-5. Medline: 34348756.
20. Mohamed AF, Isahak M, Awg Isa MZ, Nordin R. The effectiveness of workplace health promotion program in reducing work-related depression, anxiety and stress among manufacturing workers in Malaysia: mixed-model intervention. Int Arch Occup Environ Health. Jul 2022;95(5):1113-1127. doi: 10.1007/s00420-022-01836-w. Medline: 35091853.
21. Ornek OK, Esin MN. Effects of a work-related stress model based mental health promotion program on job stress, stress reactions and coping profiles of women workers: a control groups study. BMC Public Health. Nov 4, 2020;20:1658. doi: 10.1186/s12889-020-09769-0. Medline: 33148247.
22. Pandya A, Khanal N, Upadhyaya M. Workplace mental health interventions in India: a rapid systematic scoping review. Front Public Health. May 3, 2022;10:800880. doi: 10.3389/fpubh.2022.800880. Medline: 35592077.
23. Schneider RA, Grasso JR, Chen SY, Chen C, Reilly ED, Kocher B. Beyond the lab: empirically supported treatments in the real world. Front Psychol. Aug 11, 2020;11:1969. doi: 10.3389/fpsyg.2020.01969. Medline: 32849153.
24. Wan Mohd Yunus WMA, Musiat P, Brown JSL. Systematic review of universal and targeted workplace interventions for depression. Occup Environ Med. Jan 2018;75(1):66-75. doi: 10.1136/oemed-2017-104532. Medline: 29074553.
25. Xu J, Liu X, Xiao Y, Fang X, Cheng Y, Zhang J. Effect of EAP psychological intervention on improving the mental health of medical workers under the novel coronavirus epidemic in China. Front Public Health. Jul 28, 2021;9:649157. doi: 10.3389/fpubh.2021.6491572. Medline: 34395355.
26. Carolan S, Harris PR, Cavanagh K. Improving employee well-being and effectiveness: systematic review and meta-analysis of web-based psychological interventions delivered in the workplace. J Med Internet Res. Jul 26, 2017;19(7):e271. doi: 10.2196/jmir.7583. Medline: 28747293.
27. Carolan S, de Visser RO. Employees’ perspectives on the facilitators and barriers to engaging with digital mental health interventions in the workplace: qualitative study. JMIR Ment Health. Jan 19, 2018;5(1):e8. doi: 10.2196/mental.9146. Medline: 29351900.
28. Chan CMH, Ng SL, In S, Wee LH, Siau CS. Predictors of psychological distress and mental health resource utilization among employees in Malaysia. Int J Environ Res Public Health. Jan 4, 2021;18(1):314. doi: 10.3390/ijerph18010314. Medline: 33406714.
29. Dewa CS, Hoch JS. Barriers to mental health service use among workers with depression and work productivity. J Occup Environ Med. Jul 2015;57(7):726-731. doi: 10.1097/JOM.0000000000000472. Medline: 26147540.
30. Joseph B, Walker A, Fuller-Tyszkiewicz M. Evaluating the effectiveness of employee assistance programmes: a systematic review. Eur J Work Organ Psychol. Jan 2, 2018;27(1):1-15. doi: 10.1080/1359432X.2017.1374245.
31. Nunes AP, Richmond MK, Pampel FC, Wood RC. The effect of employee assistance services on reductions in employee absenteeism. J Bus Psychol. Dec 2018;33(6):699-709. doi: 10.1007/s10869-017-9518-5.
32. Peters D, Deady M, Glozier N, Harvey S, Calvo RA. Worker preferences for a mental health app within male-dominated industries: participatory study. JMIR Ment Health. Apr 25, 2018;5(2):e30. doi: 10.2196/mental.8999. Medline: 29695371.
33. Phillips EA, Gordeev VS, Schreyögg J. Effectiveness of occupational e-mental health interventions: a systematic review and meta-analysis of randomized controlled trials. Scand J Work Environ Health. Nov 1, 2019;45(6):560-576. doi: 10.5271/sjweh.3839. Medline: 31184758.
34. Scheutzow J, Attoe C, Harwood J. Acceptability of web-based mental health interventions in the workplace: systematic review. JMIR Ment Health. May 11, 2022;9(5):e34655. doi: 10.2196/34655. Medline: 35544305.
35. Smail-Crevier R, Powers G, Noel C, Wang J. Health-related internet usage and design feature preference for e-mental health programs among men and women. J Med Internet Res. Mar 18, 2019;21(3):e11224. doi: 10.2196/11224. Medline: 30882361.
36. Stratton E, Lampit A, Choi I, Calvo RA, Harvey SB, Glozier N. Effectiveness of eHealth interventions for reducing mental health conditions in employees: a systematic review and meta-analysis. PLoS One. Dec 21, 2017;12(12):e0189904. doi: 10.1371/journal.pone.0189904. Medline: 29267334.
37. van Eerd D, Cullen K, Irvin E, Le Pouésard M, Gignac M. Support for depression in the workplace: perspectives of employees, managers, and OHS personnel. Occup Health Sci. Sep 2021;5:307-343. doi:10.1007/s41542-021-00090-9.
