# Supplementary material for: A Proposed Taxonomy to Holistically Classify Employee Mental Health Programs: Qualitative Taxonomy Development Study
Source: Interact J Med Res. 2025 Dec 18;14:e67752. doi: 10.2196/67752 (PMC12746229; doi:10.2196/67752)
Supplement: Multimedia Appendix 5 [file ijmr-v14-e67752-s005.docx]

**Multimedia Appendix 5.** Relevant part of interview guide for interviews of the third iteration (translated into English given original version was in German language as interviews were conducted in Germany with German native speakers).

Context

Before the interviews begin, context on the study is provided, including the objective and approach. Further, a definition of employee mental health program (EMHP) is provided to ensure consistent understanding of what is discussed. The data privacy terms applied to this study were provided in written prior to the interviews.

Types of EMHPs

1. What types of employee health programs in general do you know?
2. What types of employee mental health programs do you know?
3. Which criteria or dimensions are relevant to categorize EMHPs?

*Other parts of the interview guide were used for another research study published by Sevov et al.*

Demographic characteristics

1. How old are you?
2. Which gender do you identify with?
3. What is your highest level of formal education?
4. How many employees work at your employer?
5. Which is the industry or sector your employer can best be assigned to?

Termination

The interview is terminated and the interviewer thanks the participant.

Note: These interviews were conducted in the context of a larger research project on EMHPs. Thus, the interviews were also used and analyzed for another study by Sevov et al., but they were analyzed differently per study as both studies had distinct research objectives. Only the above presented part of the interview guide was relevant to the present study. The full interview guide can be found in [Multimedia Appendix 2](https://jmir.org/api/download?alt_name=humanfactors_v12i1e65750_app2.docx&filename=ae722bebad23228735a2a3c1ae7a1e11.docx) of the other study (originally published in JMIR Human Factors under the terms of the [Creative Commons Attribution License 4.0](https://creativecommons.org/licenses/by/4.0/), <https://humanfactors.jmir.org/2025/1/e65750/>; Sevov B, Huettemann R, Zinner M, Meister S, Fehring L; Employee Preference and Use of Employee Mental Health Programs: Mixed Methods Study; JMIR Hum Factors 2025; 12:e65750; doi: [10.2196/65750](https://doi.org/10.2196/65750)).
