# Supplementary material for: A Proposed Taxonomy to Holistically Classify Employee Mental Health Programs: Qualitative Taxonomy Development Study
Source: Interact J Med Res. 2025 Dec 18;14:e67752. doi: 10.2196/67752 (PMC12746229; doi:10.2196/67752)
Supplement: Multimedia Appendix 6 [file ijmr-v14-e67752-s006.docx]

**Multimedia Appendix 6.** Sample of interview participants (employees) of interviews of the third iteration, N=15.

| **Variable** | **n** | **%** |
| --- | --- | --- |
| Age (y) |  |  |
| ≤29 | 3 | 20.0 |
| 30-39 | 3 | 20.0 |
| 40-49 | 3 | 20.0 |
| 50-59 | 3 | 20.0 |
| ≥60 | 3 | 20.0 |
| Gender |  |  |
| Woman | 8 | 53.3 |
| Man | 7 | 46.3 |
| Nonbinary | 0 | 0.0 |
| Education |  |  |
| None | 0 | 0.0 |
| School degree except high school (“Abitur”) | 0 | 0.0 |
| High school degree (“Abitur”) or equivalent | 0 | 0.0 |
| Professional degree, vocational training, or equivalent | 4 | 26.7 |
| Bachelor’s degree or equivalent | 2 | 13.3 |
| Master’s degree or equivalent | 8 | 53.3 |
| Doctor or PhD degree or equivalent | 1 | 6.7 |
| Number of employees |  |  |
| ≤9 | 1 | 6.7 |
| 10-49 | 2 | 13.3 |
| 50-249 | 2 | 13.3 |
| 250-499 | 1 | 6.7 |
| 500-999 | 2 | 13.3 |
| 1000-9999 | 2 | 13.3 |
| ≥10,000 | 5 | 33.3 |
| Industry |  |  |
| Energy and utilities | 1 | 6.7 |
| Raw materials and natural products | 0 | 0.0 |
| Industrial goods and services | 4 | 26.7 |
| Consumer goods and services | 2 | 13.3 |
| Commercial, technical, and scientific services as well as creative services | 1 | 6.7 |
| Health care | 2 | 13.3 |
| Financials and real estate | 1 | 6.7 |
| IT and communications | 1 | 6.7 |
| Public and governmental | 2 | 13.3 |
| Association, federation, and foundation | 1 | 6.7 |
| Other | 0 | 0.0 |

Note: These interviews were conducted in the context of a larger research project on EMHPs. Thus, the sample of interview participants of the present study was the same as that of another study published by Sevov et al. (<https://humanfactors.jmir.org/2025/1/e65750/>). Therefore, this overview of the interview participant sample is identical to the overview in [Multimedia Appendix 7](https://jmir.org/api/download?alt_name=humanfactors_v12i1e65750_app7.docx&filename=2b9ed3831d97b2399b224cad9265ed15.docx) of the other study.
