# Supplementary material for: A Proposed Taxonomy to Holistically Classify Employee Mental Health Programs: Qualitative Taxonomy Development Study
Source: Interact J Med Res. 2025 Dec 18;14:e67752. doi: 10.2196/67752 (PMC12746229; doi:10.2196/67752)
Supplement: Multimedia Appendix 7 [file ijmr-v14-e67752-s007.docx]

**Multimedia Appendix 7. Interview guide for interviews of the fourth iteration (English version – interviews were either conducted in English or German language depending on expert).**

Context

Before the interviews begin, context on the study is provided, including the objective and approach. Further, a definition of employee mental health program (EMHP) is provided to ensure consistent understanding of what is discussed. The data privacy terms applied to this study were provided in written prior to the interviews.

Discussion and confirmation of current version of taxonomy

1. Herewith, I firstly present to you the dimensions of the current taxonomy. Please get a first overview and get familiar with the dimensions. Is the structure of the taxonomy clear to you?
2. Now, we look in more detail at the characteristics of each dimension. [The following questions are discussed for each dimension]
   1. Is the presented dimension clear to you?
   2. Are the related characteristics clear to you?
   3. Which adjustments to the presented characteristics would you make?
   4. Would you merge or split specific characteristics?
   5. Would you add further characteristics, and if so, which ones?
   6. Would you change the denomination of specific characteristics, and if so, how?
   7. What other adjustments to the presented characteristics would you make?
3. Now, we take a look at the full taxonomy. Apart from the adjustments we discussed so far…
   1. … what adjustments to the presented dimensions/characteristics would you make?
   2. … would you merge or split specific dimensions/characteristics?
   3. … would you add further dimensions/characteristics?
   4. … would you change the name of specific dimensions/characteristics, and if so, how?
4. What other comments do you have on the taxonomy, how could it be further improved?

Further potential interview participants

1. Which other persons do you know who would potentially be available as experts for our study? Ideal experts are representatives of employers that offer employee mental health programs to their employees, representatives of providers of employee mental health programs, or academic key opinion leaders in that area.

Termination

We reached the end of our interview. Thank you very much for your time and expertise on this topic. Thereby, you have substantially contributed to the success of this research project.
