# Supplementary material for: A Proposed Taxonomy to Holistically Classify Employee Mental Health Programs: Qualitative Taxonomy Development Study
Source: Interact J Med Res. 2025 Dec 18;14:e67752. doi: 10.2196/67752 (PMC12746229; doi:10.2196/67752)
Supplement: Multimedia Appendix 8 [file ijmr-v14-e67752-s008.docx]

**Multimedia Appendix 8.** Sample of interview participants (experts) of interviews of the fourth iteration (N=17).

| **Variable** | **n** | **%** |
| --- | --- | --- |
| Expert group |  |  |
| Employer | 8 | 47.1 |
| Provider | 5 | 29.4 |
| Academic Key Opinion Leader (KOL) | 4 | 23.5 |
| Country |  |  |
| Germany | 5 | 29.4 |
| United States | 3 | 17.6 |
| United Kingdom | 3 | 17.6 |
| France | 2 | 11.8 |
| Japan | 2 | 11.8 |
| Sweden | 1 | 5.9 |
| Mexico | 1 | 5.9 |
| Gender |  |  |
| Female | 12 | 70.6 |
| Male | 5 | 29.4 |
| Role |  |  |
| Occupational Mental Health Expert | 4 | 23.5 |
| Occupational Health Expert | 2 | 11.8 |
| Human Resources (HR) Expert | 2 | 11.8 |
| Chief Executive Officer (CEO)/CxO/Founder | 2 | 11.8 |
| Client manager | 2 | 11.8 |
| Professor | 2 | 11.8 |
| Researcher (non-professor) | 2 | 11.8 |
| Psychological Expert | 1 | 5.9 |
