# Supplementary material for: A Proposed Taxonomy to Holistically Classify Employee Mental Health Programs: Qualitative Taxonomy Development Study
Source: Interact J Med Res. 2025 Dec 18;14:e67752. doi: 10.2196/67752 (PMC12746229; doi:10.2196/67752)
Supplement: Multimedia Appendix 9 [file ijmr-v14-e67752-s009.docx]

**Multimedia Appendix 9.** **Results of classification of “Likeminded” platform, “7Mind” app, and “Coaching & Counseling” service of “Fuerstenberg Institut” based on the new employee mental health program taxonomy, and methods and results of the interrater analysis.**

**Classification of “Likeminded” platform by 5 focus group experts**


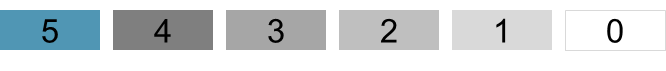

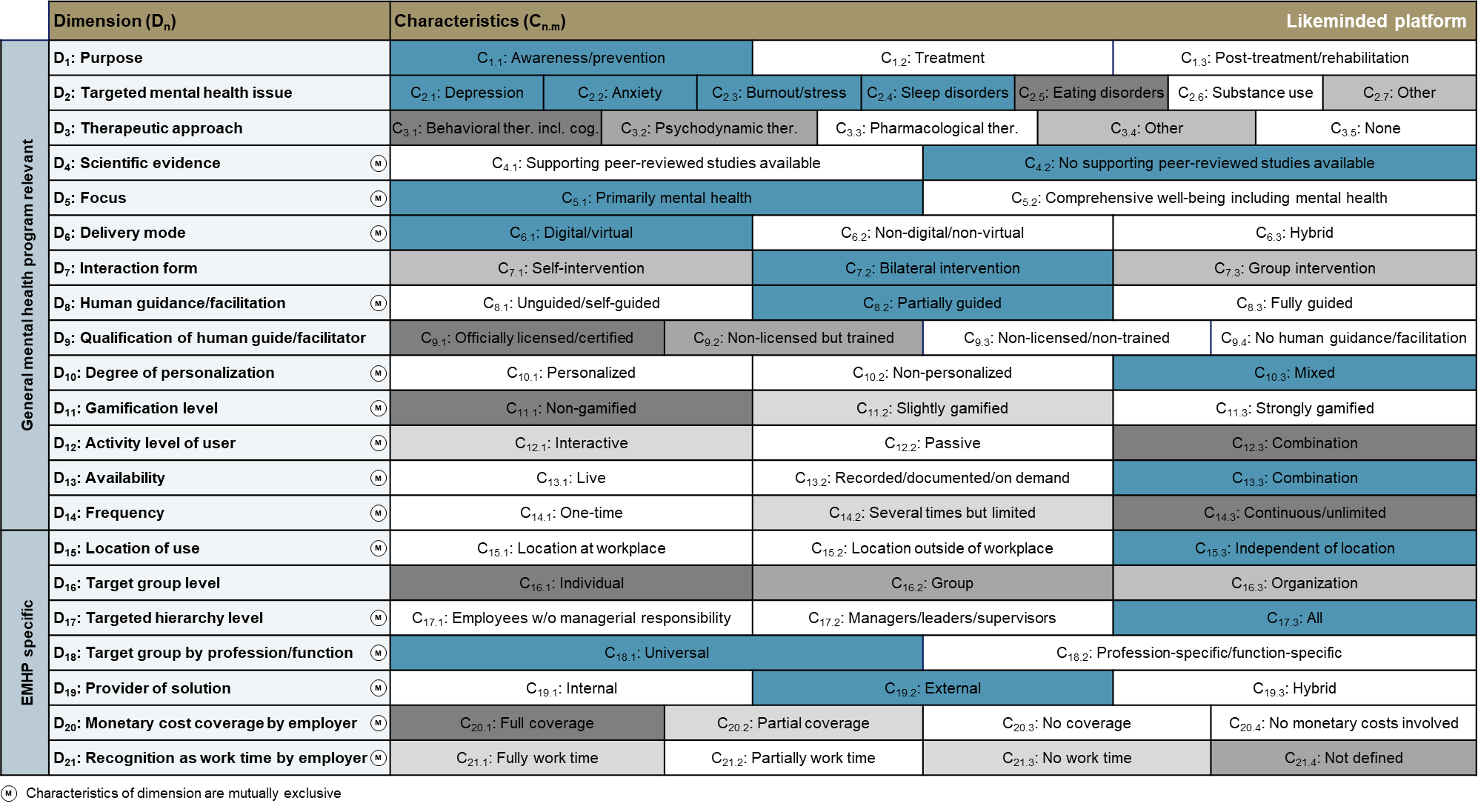


Legend for number of experts having selected each characteristic:

**Classification “7Mind” app by 5 focus group experts**


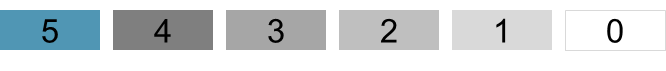

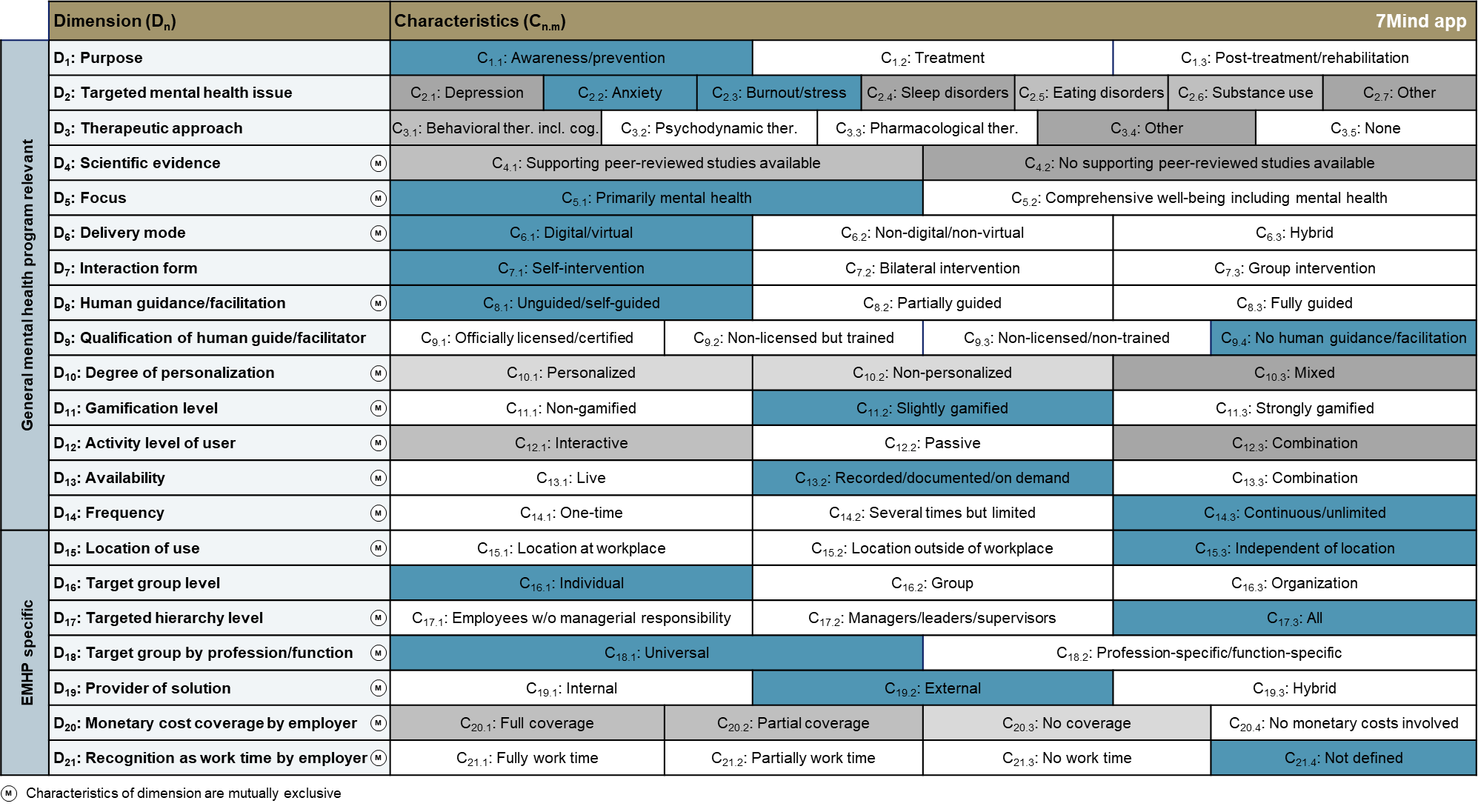


Legend for number of experts having selected each characteristic:

**Classification “Coaching & Counseling” service of “Fuerstenberg Institut” by 5 focus group experts**


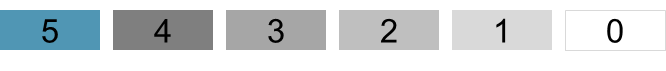

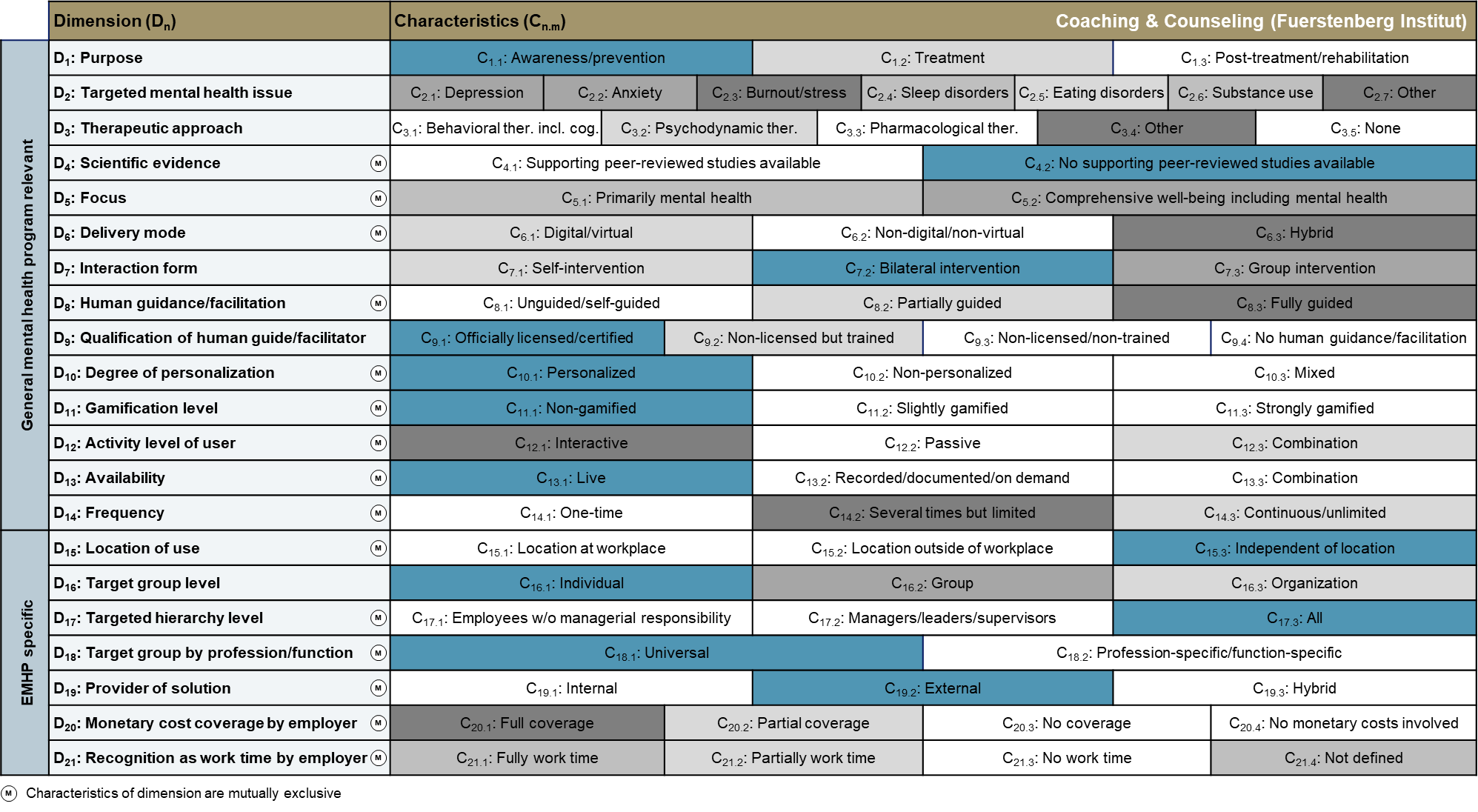


Legend for number of experts having selected each characteristic:

**Methods of interrater analysis**

We analyzed the interrater reliability among the focus group experts regarding their classifications using the measures proportion of observed agreement and Fleiss kappa (κ) [Fleiss 1971]. We used the interrater reliability approach from Fleiss as it represents an established extension of Cohen kappa that can be applied when having a constant number of more than 2 raters assessing nominal or categorical variables, which was the case in our taxonomy evaluation [Fleiss 1971, Gisev et al. 2013]. To meet the required condition of mutual exclusivity of the selectable categories, we coded the characteristics in a binary way, i.e., as 1 or 0, assessing whether the respective characteristic was selected or not by the experts [Cohen 1960]. κ ($\kappa=\frac{p_{0}- p_{e}}{1 - p_{e}}$) was calculated based on the proportion of observed agreement between the raters ($p_{0}= \frac{1}{N} \sum_{i=1}^{N} (\frac{1}{n\left( n-1 \right)} \sum_{j=1}^{k} (n_{ij}^{2}-n_{ij}))$) and the proportion of expected agreement by chance ($p_{e}= \sum_{j=1}^{k} p_{j}^{2}$) per dimension [Fleiss 1971]. n represents the number of rating experts (total of 5), N the product of the number of characteristics per dimension (ranging from 2 to 7) and the number of classified EMHPs (3), and k the number of possible outcomes, i.e., whether a rater selected a specific characteristic (binary variable with 2 possible outcomes per characteristic, 1 or 0). Ultimately, we took the unweighted average of the dimension measures to calculate the overall proportion of observed agreement and overall κ.

**Results of interrater analysis**

|  |  |  |  |  | **95% Confidence interval** | |  |
| --- | --- | --- | --- | --- | --- | --- | --- |
| **Dimension** | | **Proportion of observed agreement** | **Fleiss kappa** | **Proportion of chance agreement** | **Lower bound** | **Upper bound** | ***P* value** |
| D1 | Purpose | 0.9556 | 0.9030 | 0.5417 | 0.6964 | 1.1096 | <.001 |
| D2 | Targeted mental health issue | 0.6381 | 0.1969 | 0.5494 | 0.0616 | 0.3321 | .004 |
| D3 | Therapeutic approach | 0.7600 | 0.3656 | 0.6217 | 0.2056 | 0.5256 | <.001 |
| D4 | Scientific evidence | 0.8000 | 0.6000 | 0.5000 | 0.3470 | 0.8530 | <.001 |
| D5 | Focus | 0.8000 | 0.6000 | 0.5000 | 0.3470 | 0.8530 | <.001 |
| D6 | Delivery mode | 0.9111 | 0.8000 | 0.5556 | 0.5934 | 1.0066 | <.001 |
| D7 | Interaction form | 0.7556 | 0.5109 | 0.5002 | 0.3043 | 0.7175 | <.001 |
| D8 | Human guidance/facilitation | 0.9111 | 0.8000 | 0.5556 | 0.5934 | 1.0066 | <.001 |
| D9 | Qualification of human guide/facilitator | 0.8833 | 0.7222 | 0.5800 | 0.5433 | 0.9011 | <.001 |
| D10 | Degree of personalization | 0.8444 | 0.6500 | 0.5556 | 0.4434 | 0.8566 | <.001 |
| D11 | Gamification level | 0.9111 | 0.8000 | 0.5556 | 0.5934 | 1.0066 | <.001 |
| D12 | Activity level of user | 0.6889 | 0.3000 | 0.5556 | 0.0934 | 0.5066 | .004 |
| D13 | Availability | 1.0000 | 1.0000 | 0.5556 | 0.7934 | 1.2066 | <.001 |
| D14 | Frequency | 0.8222 | 0.6000 | 0.5556 | 0.3934 | 0.8066 | <.001 |
| D15 | Location of usage | 1.0000 | 1.0000 | 0.5556 | 0.7934 | 1.2066 | <.001 |
| D16 | Target group level | 0.7111 | 0.4219 | 0.5002 | 0.2153 | 0.6285 | <.001 |
| D17 | Targeted hierarchy level | 1.0000 | 1.0000 | 0.5556 | 0.7934 | 1.2066 | <.001 |
| D18 | Target group by profession/function | 1.0000 | 1.0000 | 0.5000 | 0.7470 | 1.2530 | <.001 |
| D19 | Provider of solution | 1.0000 | 1.0000 | 0.5556 | 0.7934 | 1.2066 | <.001 |
| D20 | Monetary cost coverage by employer | 0.7333 | 0.2889 | 0.6250 | 0.1100 | 0.4678 | .002 |
| D21 | Recognition as work time by employer | 0.7500 | 0.3333 | 0.6250 | 0.1544 | 0.5123 | <.001 |
|  | **Total** | **0.8512** | **0.6616** | **0.5523** |  |  |  |

**References:**

1. Fleiss JL. Measuring nominal scale agreement among many raters. Psychol Bull. 1971;76(5):378-382. doi: 10.1037/h0031619.
2. Gisev N, Bell JS, Chen TF. Interrater agreement and interrater reliability: key concepts, approaches, and applications. Res Social Adm Pharm. 2013;9(3):330-338. doi: 10.1016/j.sapharm.2012.04.004. Medline: 22695215.
3. Cohen J. A coefficient of agreement for nominal scales. Educ Psychol Meas. Apr 1960;20(1):37-46. doi: 10.1177/001316446002000104.
