# Supplementary material for: A Proposed Taxonomy to Holistically Classify Employee Mental Health Programs: Qualitative Taxonomy Development Study
Source: Interact J Med Res. 2025 Dec 18;14:e67752. doi: 10.2196/67752 (PMC12746229; doi:10.2196/67752)
Supplement: Multimedia Appendix 10 [file ijmr-v14-e67752-s010.docx]

**Multimedia Appendix 10. Positionality statement.**

All five authors self-identify as White Europeans and origin from a Western European country with privileged access to education and infrastructure. One author is a computer scientist and digital enthusiast. One author is a medical doctor focusing on the medical and patient perspective. Three authors are business economists focusing on the corporate perspective and practicality of health care solutions. The authors acknowledge that their personal backgrounds and privileged positions might influence their research process and interpretation of findings. To mitigate any potential influence on the research process and results, the authors ensured transparency along the whole research process and purposely included various perspectives. Different perspectives were considered as the author team itself consisted of individuals with different educational and professional backgrounds and as expert interviews with experts from 3 continents and 7 countries with different professional backgrounds were conducted.
