# Supplementary material for: A Proposed Taxonomy to Holistically Classify Employee Mental Health Programs: Qualitative Taxonomy Development Study
Source: Interact J Med Res. 2025 Dec 18;14:e67752. doi: 10.2196/67752 (PMC12746229; doi:10.2196/67752)
Supplement: Checklist 1 [file ijmr-v14-e67752-s011.docx]

**Checklist 1. The 22-item PRISMA-ScR checklist for the first iteration.**

| **Item** | **Description** | **Item reporting** |
| --- | --- | --- |
| **Section: Title** |  |  |
| 1. Title | Not applicable – scoping review only one part of overarching methodology | - |
| **Section: Abstract** |  |  |
| 2. Structured summary | **Introduction:** The number and diversity of employee mental health programs (EMHPs) has rapidly increased and is further evolving. The trend is majorly fueled by increasing need for support to sustain and regain good mental health and by technological innovation.  **Objective:** Given the large number and diversity of EMHPs, the objective was to identify relevant literature presenting taxonomies or frameworks to classify EMHPs or mental health programs in general. The existing taxonomies were intended to be used as a base for the development of the new comprehensive taxonomy to classify EMHPs.  **Methods:** A scoping review was applied to identify relevant studies presenting taxonomies and frameworks on mental health programs. The PubMed database was searched with two search queries (MeSH term and keyword search). Eligibility criteria of the pre-screening process were defined based on peer-review, abstract availability, language, and focus on taxonomy or framework. Eligibility for inclusion during the selection process based on the focus on mental health programs (Multimedia Appendix 1). The review was complemented by backward and forward snowballing.  **Results:** 17 records of relevant literature were identified, of which twelve resulted from the search and five were snowballed. From these 17 records relevant codes, i.e., dimensions and characteristics of EMHPs, were derived (Multimedia Appendix 2). Overall, 50 codes were identified and included in the taxonomy.  **Conclusion:** The scoping review represented a suitable approach to identify existing taxonomies and frameworks on mental health programs and provided relevant potential dimensions and characteristics of EMHPs, which could consequently be included in the newly developed taxonomy. | Multimedia Appendix 1, Multimedia Appendix 2 |
| **Section: Introduction** |  |  |
| 3. Rationale | As the number and diversity of employee mental health programs (EMHPs) has rapidly increased and is further evolving, established structures are useful to classify these EMHPs in the emerging landscape. Existing taxonomies and frameworks on general mental health programs can be a suitable starting point to develop a new comprehensive taxonomy on EMHPs. A scoping review seemed most appropriate to research and scope existing literature presenting such taxonomies and frameworks. Therefore, this scoping review was included as first iteration of the overall taxonomy development process of this study. | Methods |
| 4. Objectives | The scoping review aimed at identifying relevant literature presenting taxonomies or frameworks to classify EMHPs or mental health programs in general. The existing taxonomies were intended to be used as a base for the development of the new comprehensive taxonomy to classify EMHPs by directly identifying relevant dimensions and characteristics. | Methods |
| **Section: Methods** |  |  |
| 5. Protocol and registration | Not applicable | - |
| 6. Eligibility criteria | To be included in the scoping review, journal articles needed to be listed on the PubMed database. Unique articles from both search queries were included in the pre-screening process and assessed through article characteristics and title/abstract screening. Articles that were peer-reviewed, had an abstract, were in English language, and focused on a taxonomy or framework were considered for the subsequent eligibility selection process. The remaining articles were then selected based on full-text assessment during the eligibility selection process based on the focus of the presented taxonomy or framework (Multimedia Appendix 1). | Multimedia Appendix 1 |
| 7. Information sources | The PubMed database was searched to conduct the scoping review. The search queries were performed on 2 January 2024. | Methods |
| 8. Search | The search was conducted by BS with defined search queries. Initial article searches were conducted to identify relevant articles and derive established and widely-used terms and MeSH terms. These identified terms were then discussed within the entire author team – including SM and LF, who have extensive experience conducting scoping reviews and have published more than 20 peer-reviewed articles – before we executed the final searches in two streams. First, combinations of defined MeSH terms were used to search the database:  (1) ((taxonomy[MeSH Terms]) OR (framework[MeSH Terms]) OR (structure[MeSH Terms]) OR (classification[MeSH Terms]) OR (categorization[MeSH Terms])) AND ((mental health intervention[MeSH Terms]) OR (mental health program[MeSH Terms]))  Second, defined keywords were searched for in the articles’ titles and abstracts:  (2) ((taxonomy[Title/Abstract]) OR (framework[Title/Abstract]) OR (structure[Title/Abstract]) OR (classification[Title/Abstract]) OR (categorization[Title/Abstract])) AND ((mental health intervention*[Title/Abstract]) OR (mental health program*[Title/Abstract]))  The MeSH terms and keywords were selected such that the search would yield articles on relevant taxonomies or frameworks, which would in some way represent a suitable structure to classify general mental health programs.  No librarian/information specialist consulted the search. However, it was carefully adhered to general scoping review guidelines, especially the PRISMA-ScR guideline, and two authors, SM and LF, were sufficiently experienced with conducting scoping reviews. | Methods |
| 9. Selection of sources of evidence | A two-step screening rationale was applied to identify the relevant articles. First, the found articles were pre-screened through article characteristics and title/abstract screening regarding peer-review, abstract availability, language, and focus on a taxonomy or framework. Second, the remaining articles were selected through a full-text assessment based on focus of the taxonomy or framework (Multimedia Appendix 1). | Multimedia Appendix 1 |
| 10. Data charting process | All included and snowballed articles were recorded in a tabular overview including title, DOI, authors, journal, year of publication, research focus, and derived codes for the newly developed taxonomy (Multimedia Appendix 2). The ‘derived codes’ indicate which dimensions and characteristics were included in the new taxonomy on EMHPs. The texts of the articles were systematically screened by two authors, BS and RH, through two separate coding rounds and the codes were assigned to the respective parts of the text, i.e., the presented taxonomies or frameworks, representing relevant potential dimensions and characteristics of EMHPs. | Multimedia Appendix 2 |
| 11. Data items | All included and snowballed articles were screened for the taxonomy or framework on mental health programs and the presented potential dimensions and characteristics were coded. The final codes were used for inclusion in the newly developed taxonomy (Multimedia Appendix 2). | Multimedia Appendix 2 |
| 12. Critical appraisal of individual sources of evidence | Given the articles included in the review were used to derive potential dimensions and characteristics of EMHPs to inform the development of the new taxonomy on EMHPs, no risk of bias was expected. The findings represented only one of four iterations of the applied taxonomy development process. | - |
| 13. Synthesis of results | All articles included in the review and all snowballed articles were recorded in a tabular overview including title, DOI, authors, journal, year of publication, research focus, and derived codes for the newly developed taxonomy (Multimedia Appendix 2). | Multimedia Appendix 2 |
| **Section: Results** |  |  |
| 14. Selection of sources of evidence | After removal of duplicates, the two search queries resulted in 446 articles included in the pre-screening process. After pre-screening through assessing article characteristics and title/abstract review, 124 articles were assessed for eligibility through full-text assessment. After careful eligibility selection, twelve articles were included in the review. The details of the selection process including the reasons for exclusion can be found in Multimedia Appendix 1. Backward and forward snowballing was conducted leading to five further identified articles. | Multimedia Appendix 1 |
| 15. Characteristics of sources of evidence | All included and snowballed articles were recorded in a tabular overview including title, DOI, authors, journal, year of publication, research focus, and derived codes for the newly developed taxonomy (Multimedia Appendix 2). The ‘derived codes’ indicate which dimensions and characteristics were included in the new taxonomy on EMHPs. | Multimedia Appendix 2 |
| 16. Critical appraisal within sources of evidence | Given the articles included in the review were used to derive potential dimensions and characteristics of EMHPs to inform the development of the new taxonomy on EMHPs, no risk of bias was expected. The findings represented only one of four iterations of the applied taxonomy development process. | - |
| 17. Results of individual sources of evidence | The derived codes per article used for the development of the new taxonomy are presented in Multimedia Appendix 2. | Multimedia Appendix 2 |
| 18. Synthesis of results | A tabular overview of the included and snowballed articles informs about the derived codes (Multimedia Appendix 2). | Multimedia Appendix 2 |
| **Section: Discussion** |  |  |
| 19. Summary of evidence | The scoping review revealed twelve relevant articles presenting taxonomies or frameworks to classify mental health programs. Five articles were identified through snowballing. The findings of the articles were used to inform the development of the new taxonomy on EMHPs (Multimedia Appendix 2). | Multimedia Appendix 2 |
| 20. Limitations | While the PubMed database was carefully chosen for the scoping review and represents a reliable source of scientific articles in the medical/healthcare area, scoping reviews could search several databases to include as many potential articles as possible. By searching only one database, relevant articles might have potentially been missed. However, through snowballing, other relevant articles were considered and included in the review. Further, this scoping review represented only one of four iterations of the applied taxonomy development process. | - |
| 21. Conclusions | The findings and 50 derived codes of the included and snowballed articles led to a relevant part of dimensions and characteristics included in the newly developed taxonomy. The review also showed that only very few taxonomies or frameworks are available to classify mental health programs, of which most only focus on specific aspects, and merely any on classifying EMHPs. This research gap was addressed with the present study by developing a comprehensive taxonomy to classify EMHPs. | Introduction; Results |
| **Section: Funding** |  |  |
| 22. Funding | No funding was received for this research. | Declarations |

Based on: Tricco AC, Lillie E, Zarin W, et al; PRISMA extension for scoping reviews (PRISMA-ScR): checklist and explanation; Ann Intern Med 2018; 169(7): 467–473; doi: [10.7326/M18-0850](https://doi.org/10.7326/m18-0850).
