# Supplementary material for: A Proposed Taxonomy to Holistically Classify Employee Mental Health Programs: Qualitative Taxonomy Development Study
Source: Interact J Med Res. 2025 Dec 18;14:e67752. doi: 10.2196/67752 (PMC12746229; doi:10.2196/67752)
Supplement: Checklist 2 [file ijmr-v14-e67752-s012.docx]

**Checklist 2. The 22-item PRISMA-ScR checklist for the second iteration.**

| **Item** | **Description** | **Item reporting** |
| --- | --- | --- |
| **Section: Title** |  |  |
| 1. Title | Not applicable – scoping review only one part of overarching methodology | - |
| **Section: Abstract** |  |  |
| 2. Structured summary | **Introduction:** Different employee mental health programs (EMHPs) are deployed to support employee mental health. Various research exists that investigates on different aspects of EMHPs. All these research studies reveal information on different types of EMHPs, which are helpful to develop a comprehensive taxonomy on EMHPs.  **Objective:** Given the large number and diversity of EMHPs, the objective was to identify relevant literature on EMHPs, which could inform the identification of relevant characteristics of EMHPs by which these programs can be described and classified. These characteristics were intended to be used as a base for the development of the new comprehensive taxonomy to classify EMHPs.  **Methods:** A scoping review was applied to identify relevant studies on EMHPs. The PubMed database was searched with two search queries. Eligibility criteria were defined around year of publication, language, abstract availability, and different content-related criteria (Multimedia Appendix 3). The review was complemented by backward and forward snowballing.  **Results:** 25 records of relevant literature were identified. From these and twelve further records identified through snowballing (Multimedia Appendix 3), codes representing relevant potential dimensions and characteristics of EMHPs were derived (Multimedia Appendix 4). Overall, 64 codes were identified and included in the taxonomy.  **Conclusion:** The scoping review was an adequate approach to identify relevant studies on EMHPs stating several potential dimensions and characteristics of EMHPs, which could consequently be included in the newly developed taxonomy. | Multimedia Appendix 3, Multimedia Appendix 4 |
| **Section: Introduction** |  |  |
| 3. Rationale | As the number and diversity of employee mental health programs (EMHPs) has rapidly increased and is further evolving, established structures are useful to classify these EMHPs in the emerging landscape. Existing research on various aspects of EMHPs represents a solid base for identifying relevant dimensions and characteristics of EMHPs. Therefore, a scoping review seemed most appropriate to research and scope existing literature on EMHPs, including meta-reviews, which list many different EMHPs. Thus, this scoping review was included as second iteration of the overall taxonomy development process of this study. This scoping review was conducted in the context of a larger research project on EMHPs. Thus, the identified literature records of this review were also used and analyzed for another study. However, the identified literature records were analyzed differently per study as both studies had distinct research objectives. | Methods |
| 4. Objectives | The scoping review aimed at identifying relevant literature on EMHPs to derive relevant dimensions and characteristics of EMHPs, which were intended to be used as a base for the development of the new comprehensive taxonomy to classify EMHPs. | Methods |
| **Section: Methods** |  |  |
| 5. Protocol and registration | Not applicable | - |
| 6. Eligibility criteria | To be included in the scoping review, journal articles needed to be listed on the PubMed database. Unique articles from both search queries were included in the pre-screening process, which assessed the articles based on filters and technical criteria. Articles published between January 1, 2018 and including 11 January 2023, in English language, and providing an abstract were considered for the subsequent eligibility selection process. The publication period of five years from 2018 to beginning 2023 was chosen to only include current research ensuring that newer EMHPs, including the ones leveraging technology, were considered. The remaining articles were then selected based on full-text assessment during the eligibility selection process based on specific content-related eligibility criteria (Multimedia Appendix 3). | Multimedia Appendix 3 |
| 7. Information sources | The PubMed database was searched to conduct the scoping review. The search queries were performed on 12 January 2023, while the literature records were analyzed in January 2024. | Methods |
| 8. Search | The search was conducted by BS with defined search queries. Initial article searches were conducted to identify relevant articles and derive established and widely-used keywords and MeSH terms. These identified terms were then discussed within the entire author team – including SM and LF, who have extensive experience conducting scoping reviews and have published more than 20 peer-reviewed articles – before we executed the final searches in two streams. First, combinations of defined MeSH terms were used to search the database:  (1) ((mental health[MeSH Terms]) OR (psychology[MeSH Terms])) AND ((employee assistance program[MeSH Terms]) OR (employer intervention[MeSH Terms]) OR (workplace intervention[MeSH Terms])) OR ((mental health[MeSH Terms]) AND (workplace[MeSH Terms]) AND (health promotion[MeSH Terms]))  Second, defined keywords were searched for in the articles’ titles and abstracts:  (2) (mental health[Title/Abstract]) AND ((employee assistance program[Title/Abstract]) OR (employer intervention[Title/Abstract]) OR (workplace intervention[Title/Abstract]))  The MeSH terms and keywords were selected such that the search would yield articles on any program or intervention addressing mental health at the workplace.  No librarian/information specialist consulted the search. However, it was carefully adhered to general scoping review guidelines, especially the PRISMA-ScR guideline, and two authors, SM and LF, were sufficiently experienced with conducting scoping reviews. | Methods |
| 9. Selection of sources of evidence | A two-step screening rationale was applied to identify the relevant articles. First, the found articles were pre-screened through filters and review of technical criteria regarding publication date, language, and abstract availability. Second, the remaining articles were selected through a full-text assessment based on the defined content-related eligibility criteria (Multimedia Appendix 3). | Multimedia Appendix 3 |
| 10. Data charting process | All included and snowballed articles were recorded in a tabular overview including title, DOI, authors, journal, year of publication, research focus, and derived codes for the newly developed taxonomy (Multimedia Appendix 4). The ‘derived codes’ indicate which dimensions and characteristics were included in the new taxonomy on EMHPs. The texts of the articles were systematically screened by two authors, BS and RH, through two separate coding rounds and the codes were assigned to the respective parts of the text, i.e., the identified studies, representing relevant potential dimensions and characteristics of EMHPs. | Multimedia Appendix 4 |
| 11. Data items | All included and snowballed articles were screened for parts of the text representing relevant potential dimensions and characteristics of EMHPs. These parts were coded and the resulting final codes were considered for inclusion in the newly developed taxonomy (Multimedia Appendix 4). | Multimedia Appendix 4 |
| 12. Critical appraisal of individual sources of evidence | Given the articles included in the review were used to derive potential dimensions and characteristics of EMHPs to inform the development of the new taxonomy on EMHPs, no risk of bias was expected. The findings represented only one of four iterations of the applied taxonomy development process. | - |
| 13. Synthesis of results | All articles included in the review and all snowballed articles were recorded in a tabular overview including title, DOI, authors, journal, year of publication, research focus, and derived codes for the newly developed taxonomy (Multimedia Appendix 4). | Multimedia Appendix 4 |
| **Section: Results** |  |  |
| 14. Selection of sources of evidence | After removal of duplicates, the two search queries resulted in 429 articles included in the pre-screening process. After pre-screening through filters and abstract review, 115 articles were assessed for eligibility through full-text assessment. After careful eligibility selection, 25 articles were included in the review. The details of the selection process including the reasons for exclusion can be found in Multimedia Appendix 3. Backward and forward snowballing was conducted leading to twelve identified articles. | Multimedia Appendix 3 |
| 15. Characteristics of sources of evidence | All included and snowballed articles were recorded in a tabular overview including title, DOI, authors, journal, year of publication, research focus, and derived codes for the newly developed taxonomy (Multimedia Appendix 4). The ‘derived codes’ indicate which dimensions and characteristics were included in the new taxonomy on EMHPs. | Multimedia Appendix 4 |
| 16. Critical appraisal within sources of evidence | Given the articles included in the review were used to derive potential dimensions and characteristics of EMHPs to inform the development of the new taxonomy on EMHPs, no risk of bias was expected. The findings represented only one of four iterations of the applied taxonomy development process. | - |
| 17. Results of individual sources of evidence | The derived codes per article used for the development of the new taxonomy are presented in Multimedia Appendix 4. | Multimedia Appendix 4 |
| 18. Synthesis of results | A tabular overview of the included and snowballed articles informs about the derived codes (Multimedia Appendix 4). | Multimedia Appendix 4 |
| **Section: Discussion** |  |  |
| 19. Summary of evidence | The scoping review revealed 25 relevant articles published between 2018 and 2023 researching different aspects of EMHPs. Twelve articles were identified through snowballing. The findings of the articles were used to inform the development of the new taxonomy on EMHPs (Multimedia Appendix 4). | Multimedia Appendix 4 |
| 20. Limitations | While the PubMed database was carefully chosen for the scoping review and represents a reliable source of scientific articles in the medical/healthcare area, scoping reviews could search several databases to include as many potential articles as possible. By searching only one database, relevant articles might have potentially been missed. However, through snowballing, other relevant articles were considered and included in the review. Further, this scoping review represented only one of four iterations of the applied taxonomy development process. | - |
| 21. Conclusions | The 64 derived codes of the included and snowballed articles led to a relevant part of dimensions and characteristics included in the newly developed taxonomy. The review also emphasized the increasing importance of EMHPs and the increasing focus of research on EMHPs and related aspects. These findings reasonably imply the need for more transparency on the EMHP landscape. | Introduction; Results |
| **Section: Funding** |  |  |
| 22. Funding | No funding was received for this research. | Declarations |

Based on: Tricco AC, Lillie E, Zarin W, et al; PRISMA extension for scoping reviews (PRISMA-ScR): checklist and explanation; Ann Intern Med 2018; 169(7): 467–473; doi: [10.7326/M18-0850](https://doi.org/10.7326/m18-0850).
