# Supplementary material for: A Proposed Taxonomy to Holistically Classify Employee Mental Health Programs: Qualitative Taxonomy Development Study
Source: Interact J Med Res. 2025 Dec 18;14:e67752. doi: 10.2196/67752 (PMC12746229; doi:10.2196/67752)
Supplement: Checklist 3 [file ijmr-v14-e67752-s013.docx]

**Checklist 3. The 32-item COREQ checklist for interviews of the third iteration.**

| **Item** | **Guide question** | **Description** | **Item reporting** |
| --- | --- | --- | --- |
| **Domain 1: Research team and reflexivity** | | | |
| *Personal characteristics* | | | |
| 1. Interviewer/facilitator | Which author/s conducted the interview or focus group? | BS conducted the interviews | - |
| 2. Credentials | What were the researcher’s credentials? E.g., PhD, MD | BS holds a BA and two MSc | - |
| 3. Occupation | What was their occupation at the time of the study? | BS is a PhD candidate at the Witten/Herdecke University | Title page |
| 4. Gender | Was the researcher male or female? | BS is male | - |
| 5. Experience and training | What experience or training did the researcher have? | BS has experience in conducting qualitative interviews through interview studies during MSc program | - |
| *Relationship with participants* | | | |
| 6. Relationship established | Was a relationship established prior to study commencement? | Participants were recruited from the broader network of all authors; therefore, the interviewer knew some of the participants by name and demographic characteristics prior to the study | - |
| 7. Participant knowledge of the interviewer | What did the participants know about the researcher? E.g., personal goals, reasons for doing the research | All participants were briefed on the objective of the conducted research and were provided with the relevant information material (i.e., privacy statement); when asked, the researcher explained that the study was conducted in the context of a PhD program | Methods |
| 8. Interviewer characteristics | What characteristics were reported about the interviewer/ facilitator? E.g., Bias, assumptions, reasons and interests in the research topic | No characteristics on the interviewer were reported as no relevant biases were expected; the interviewer only had scientific interest in the study, there was no economic interest | - |
| **Domain 2: Study design** | | | |
| *Theoretical framework* | | | |
| 9. Methodological orientation and theory | What methodological orientation was stated to underpin the study? E.g., grounded theory, discourse analysis, ethnography, phenomenology, content analysis | Thematic analysis was applied through two coding rounds | Methods |
| *Participant selection* | | | |
| 10. Sampling | How were participants selected? E.g., purposive, convenience, consecutive, snowball | A purposive sampling was applied; participants were recruited from the broader network of all authors and selected such that a representative sample of the observed population (employees of companies in Germany) was obtained, distributed across demographic and company characteristics (e.g., age, gender, industry of employer) | Methods |
| 11. Method of approach | How were participants approached? E.g., face-to-face, telephone, mail, email | Participants were contacted via email | - |
| 12. Sample size | How many participants were in the study? | 15 participants were interviewed | Results |
| 13. Non-participation | How many people refused to participate or dropped out? Reasons? | All contacted persons were willing to participate in the interview study, none of them dropped out during the interview process | - |
| *Setting* | | | |
| 14. Setting of data collection | Where was the data collected? E.g., home, clinic, workplace | Data was collected either in personal settings in private residence rooms of the participants or via phone/video calls in February and March 2023 | - |
| 15. Presence on non-participants | Was anyone else present besides the participants and researchers? | No, only BS and the respective participant were present | - |
| 16. Description of sample | What are the important characteristics of the sample? E.g., demographic data, date | The sample was well distributed across relevant defined characteristics, i.e., age, gender, education, number of employees, industry of employer | Multimedia Appendix 6 |
| *Data collection* | | | |
| 17. Interview guide | Were questions, prompts, guides provided by the authors? Was it pilot tested? | Interviews were semi-structured based on a prepared interview guide (Multimedia Appendix 5) which was tested and iterated by co-authors; interviews contributed to two research studies that form part of a larger research project, only questions 1. to 3. and 11. to 15. were relevant for this study | Methods; Multimedia Appendix 5 |
| 18. Repeat interviews | Were repeat interviews carried out? If yes, how many? | No repeat interviews were required | - |
| 19. Audio/visual recording | Did the research use audio or visual recording to collect the data? | Interviews, both, face-to-face and via phone/video call, were audio recorded | - |
| 20. Field notes | Were field notes made during and/or after the interview or focus group? | No field notes were made, the audio recordings were transcribed after the interviews | - |
| 21. Duration | What was the duration of the interviews or focus group? | Interview durations ranged from 25 to 46 minutes | - |
| 22. Data saturation | Was data saturation discussed? | Data saturation was not discussed with participants | - |
| 23. Transcripts returned | Were transcripts returned to participants for comment and/or correction? | Transcripts were not returned to participants | - |
| **Domain 3: Analysis and findings** | | | |
| *Data analysis* | | | |
| 24. Number of data coders | How many data coders coded the data? | Two authors, BS and RH, coded and reviewed the data independently | Methods |
| 25. Description of the coding tree | Did authors provide a description of the coding tree? | Thematic analysis was conducted, the interview transcripts were coded in two coding rounds (first round: initial coding, second round: double-check and aggregation of codes) | Methods |
| 26. Derivation of themes | Were themes identified in advance or derived from the data? | Themes were derived in advance from scoping reviews; however, additional themes were derived from the data | Methods |
| 27. Software | What software, if applicable, was used to manage the data? | MaxQDA was used to manage and analyze the data; Microsoft Word was used to create the transcripts | Methods |
| 28. Participant checking | Did participants provide feedback on the findings? | No | - |
| *Reporting* | | | |
| 29. Quotations presented | Were participant quotations presented to illustrate the themes/findings?  Was each quotation identified? E.g., participant number | No quotations were presented in the manuscript to support the findings | - |
| 30. Data and findings consistent | Was there consistency between the data presented and the findings? | Study findings were reported such that they are consistent with the collected data | - |
| 31. Clarity of major themes | Were major themes clearly presented in the findings? | Major themes informed the development of the initial version of the taxonomy; 55 codes were derived from analyzing the interview transcripts and used in the taxonomy; further, statements of interview participants informed parts of the reasonings in the findings | - |
| 32. Clarity of minor themes | Is there a description of diverse cases or discussion of minor themes? | Not applicable | - |

Based on: Tong A, Sainsbury P, Craig J; Consolidated criteria for reporting qualitative research (COREQ): a 32-item checklist for interviews and focus groups; Int J Qual Health Care 2007; 19(6): 349–357; doi: [10.1093/intghq/mzm042](https://doi.org/10.1093/intqhc/mzm042).
