# Supplementary material for: A Proposed Taxonomy to Holistically Classify Employee Mental Health Programs: Qualitative Taxonomy Development Study
Source: Interact J Med Res. 2025 Dec 18;14:e67752. doi: 10.2196/67752 (PMC12746229; doi:10.2196/67752)
Supplement: Checklist 4 [file ijmr-v14-e67752-s014.docx]

**Checklist 4. The 32-item COREQ checklist for interviews of the fourth iteration.**

| **Item** | **Guide question** | **Description** | **Item reporting** |
| --- | --- | --- | --- |
| **Domain 1: Research team and reflexivity** | | | |
| *Personal characteristics* | | | |
| 1. Interviewer/facilitator | Which author/s conducted the interview or focus group? | BS conducted the interviews | - |
| 2. Credentials | What were the researcher’s credentials? E.g., PhD, MD | BS holds a BA and two MSc | - |
| 3. Occupation | What was their occupation at the time of the study? | BS is a PhD candidate at the Witten/Herdecke University | Title page |
| 4. Gender | Was the researcher male or female? | BS is male | - |
| 5. Experience and training | What experience or training did the researcher have? | BS has experience in conducting qualitative interviews through interview studies during MSc program | - |
| *Relationship with participants* | | | |
| 6. Relationship established | Was a relationship established prior to study commencement? | Participants were recruited based on the pre-defined systematic sampling approach (described in the Methods part), therefore, no relationship was established prior to the study | Methods |
| 7. Participant knowledge of the interviewer | What did the participants know about the researcher? E.g., personal goals, reasons for doing the research | All participants were briefed on the objective of the conducted research and were provided with the relevant information material (i.e., privacy statement); participants were informed that the study was conducted in the context of a PhD program | Methods |
| 8. Interviewer characteristics | What characteristics were reported about the interviewer/ facilitator? E.g., Bias, assumptions, reasons and interests in the research topic | No characteristics on the interviewer were reported as no relevant biases were expected; the interviewer only had scientific interest in the study, there was no economic interest | - |
| **Domain 2: Study design** | | | |
| *Theoretical framework* | | | |
| 9. Methodological orientation and theory | What methodological orientation was stated to underpin the study? E.g., grounded theory, discourse analysis, ethnography, phenomenology, content analysis | The overarching methodology, i.e., the taxonomy development process, was based on a well-established taxonomy development approach from Nickerson et al. (2013) and Kundisch et al. (2022); the interview study was conducted as the fourth of four iterations in the taxonomy development process to refine and validate an initial version of the developed taxonomy; adjustments to the respective taxonomy version were discussed during the interviews and executed directly after each interview | Methods |
| *Participant selection* | | | |
| 10. Sampling | How were participants selected? E.g., purposive, convenience, consecutive, snowball | Three expert groups were considered, for each expert group a systematic sampling approach was applied;  Employers: employers were identified through two keyword searches on LinkedIn (I. “employee mental health”, II. “occupational mental health”) and directly contacted via the platform; the keyword search was performed five times, each time limiting the search to experts located in one of the five defined countries Germany, United Kingdom, France, United Stated of America, and Japan;  Providers: providers were identified through a Google keyword search for each of the five countries (I. English term for all countries: “employee mental health program provider COUNTRY”, plus II: local language term for Germany “anbieter mitarbeiterprogramm mentale gesundheit” and for France “fournisseur programme santé mentale des employés france”); representatives of the providers were contacted via publicly available contact data;  Academic Key Opinion Leaders (KOLs): KOLs were identified based on the number of publications on employee mental health topics; relevant publications were identified through a keyword search on the ‘PubMed’ database (“(employee mental  health[Title/Abstract]) OR (employee mental health program[Title/Abstract]) OR (employee assistance program[Title/Abstract])”), KOLs were contacted via publicly available email address | Methods |
| 11. Method of approach | How were participants approached? E.g., face-to-face, telephone, mail, email | Employers were contacted via LinkedIn, providers were contacted via email or LinkedIn, academic KOLs were contacted via email; in total, 73 experts were contacted, thereof 43 employers, 20 providers, and 10 KOLs | Methods |
| 12. Sample size | How many participants were in the study? | 17 experts were interviewed | Methods |
| 13. Non-participation | How many people refused to participate or dropped out? Reasons? | A relevant share of contacted experts did not reply to LinkedIn messages or emails; some contacted experts replied but were not available for an expert interview due to lack of time; of the participating experts, no one dropped out once they confirmed their participation and started the interview | - |
| *Setting* | | | |
| 14. Setting of data collection | Where was the data collected? E.g., home, clinic, workplace | Data was collected via video calls through January to April 2024 |  |
| 15. Presence on non-participants | Was anyone else present besides the participants and researchers? | No, only BS and the respective participant were present | - |
| 16. Description of sample | What are the important characteristics of the sample? E.g., demographic data, date | The sample was well distributed across the three defined expert groups and the five selected countries and further countries (received through snowballing of experts based on recommendation of interviewed experts) | Multimedia Appendix 8 |
| *Data collection* | | | |
| 17. Interview guide | Were questions, prompts, guides provided by the authors? Was it pilot tested? | Interviews were semi-structured based on a prepared interview guide (Multimedia Appendix 7) | Methods; Multimedia Appendix 7 |
| 18. Repeat interviews | Were repeat interviews carried out? If yes, how many? | No repeat interviews were required | - |
| 19. Audio/visual recording | Did the research use audio or visual recording to collect the data? | Interviews were audio recorded | - |
| 20. Field notes | Were field notes made during and/or after the interview or focus group? | Field notes were made during the interviews to document the discussed adjustments to the taxonomy; the audio recordings were transcribed after the interviews for documentation and review purposes | - |
| 21. Duration | What was the duration of the interviews or focus group? | Interview durations ranged from 22 to 65 minutes | - |
| 22. Data saturation | Was data saturation discussed? | Data saturation was not discussed with participants | - |
| 23. Transcripts returned | Were transcripts returned to participants for comment and/or correction? | Transcripts were not returned to participants | - |
| **Domain 3: Analysis and findings** | | | |
| *Data analysis* | | | |
| 24. Number of data coders | How many data coders coded the data? | The interview transcripts were not coded as adjustments to the respective taxonomy version were concretely discussed during the interviews (see for instance Scheider et al., 2023, Exploring Design Elements of Personal Data Markets - A Taxonomy and  Archetypes); the interview transcripts were produced for documentation purposes and for review by the authors after closing of the interview study | - |
| 25. Description of the coding tree | Did authors provide a description of the coding tree? | Not applicable | - |
| 26. Derivation of themes | Were themes identified in advance or derived from the data? | Not applicable | - |
| 27. Software | What software, if applicable, was used to manage the data? | Microsoft Word was used to create the transcripts | - |
| 28. Participant checking | Did participants provide feedback on the findings? | No | - |
| *Reporting* | | | |
| 29. Quotations presented | Were participant quotations presented to illustrate the themes/findings?  Was each quotation identified? E.g., participant number | No quotations were presented in the manuscript to support the findings | - |
| 30. Data and findings consistent | Was there consistency between the data presented and the findings? | Study findings, i.e., the taxonomy, were reported such that they are consistent with the collected data, i.e., the concrete expert input on how to adjust the taxonomy | - |
| 31. Clarity of major themes | Were major themes clearly presented in the findings? | Not applicable | - |
| 32. Clarity of minor themes | Is there a description of diverse cases or discussion of minor themes? | Not applicable | - |

Based on: Tong A, Sainsbury P, Craig J; Consolidated criteria for reporting qualitative research (COREQ): a 32-item checklist for interviews and focus groups; Int J Qual Health Care 2007; 19(6): 349–357; doi: [10.1093/intghq/mzm042](https://doi.org/10.1093/intqhc/mzm042).
