# Supplementary material for: A Proposed Taxonomy to Holistically Classify Employee Mental Health Programs: Qualitative Taxonomy Development Study
Source: Interact J Med Res. 2025 Dec 18;14:e67752. doi: 10.2196/67752 (PMC12746229; doi:10.2196/67752)
Supplement: Checklist 5 [file ijmr-v14-e67752-s015.docx]

**Checklist 5. The 32-item COREQ checklist for the focus group.**

| **Item** | **Guide question** | **Description** | **Item reporting** |
| --- | --- | --- | --- |
| **Domain 1: Research team and reflexivity** | | | |
| *Personal characteristics* | | | |
| 1. Interviewer/facilitator | Which author/s conducted the interview or focus group? | BS moderated the focus group; LF co-moderated the focus group | - |
| 2. Credentials | What were the researcher’s credentials? E.g., PhD, MD | BS holds a BA and two MSc | - |
| 3. Occupation | What was their occupation at the time of the study? | BS is a PhD candidate at the Witten/Herdecke University | Title page |
| 4. Gender | Was the researcher male or female? | BS is male | - |
| 5. Experience and training | What experience or training did the researcher have? | BS has experience in conducting qualitative interviews through interview studies during MSc program; BS prepared the focus group session together with other researchers who had conducted focus groups before | - |
| *Relationship with participants* | | | |
| 6. Relationship established | Was a relationship established prior to study commencement? | The focus group experts were selected based on their expertise and their relationship with the selected EMHPs to which the taxonomy should be applied; one of the experts was part of the broader network of the authors; no relationship to the other experts was established prior to the study | - |
| 7. Participant knowledge of the interviewer | What did the participants know about the researcher? E.g., personal goals, reasons for doing the research | All participants were briefed on the objective of the conducted research and were provided with the relevant information material (i.e., privacy statement); participants were informed that the study was conducted in the context of a PhD program | - |
| 8. Interviewer characteristics | What characteristics were reported about the interviewer/ facilitator? E.g., Bias, assumptions, reasons and interests in the research topic | No further characteristics on the two moderators, apart from the focus of the research, were reported as no relevant biases were expected; the moderators only had scientific interest in the study, there was no economic interest | - |
| **Domain 2: Study design** | | | |
| *Theoretical framework* | | | |
| 9. Methodological orientation and theory | What methodological orientation was stated to underpin the study? E.g., grounded theory, discourse analysis, ethnography, phenomenology, content analysis | Taxonomy evaluation through a focus group with experts (see Szopinski et al., 2019, Because Your Taxonomy is Worth It: Towards a Framework for Taxonomy Evaluation); one EMHP was independently classified during the focus group session; subsequently, the classification was discussed by the focus group experts; the classifications of the remaining two EMHPs were performed individually and independently by the focus group experts in written form and collected by the authors (as described in the Methods part); the transcript was analyzed by BS and LF; the classifications were analyzed by BS; LF reviewed the analysis | Methods |
| *Participant selection* | | | |
| 10. Sampling | How were participants selected? E.g., purposive, convenience, consecutive, snowball | Purposive sampling was applied; participants were recruited based on their expertise and their relationship with the selected EMHPs; participants were selected such that one representative of each of the three classified EMHPs participated, to ensure adequate program-specific insight, and that they were not involved as an expert in the fourth iteration | Methods |
| 11. Method of approach | How were participants approached? E.g., face-to-face, telephone, mail, email | Participants were contacted via email or LinkedIn | - |
| 12. Sample size | How many participants were in the study? | Five experts participated in the focus group | Methods |
| 13. Non-participation | How many people refused to participate or dropped out? Reasons? | Some contacted experts refused to participate in the focus group due to lack of time; of the participating experts, no one dropped out once they confirmed their participation and joined the focus group; all participating experts provided the offline classifications of the two remaining EMHPs | - |
| *Setting* | | | |
| 14. Setting of data collection | Where was the data collected? E.g., home, clinic, workplace | Data was collected via a video call using the Zoom software; the focus group was conducted in German language as all participants were fluent in German; the classifications of the two remaining EMHPs were collected in written form | - |
| 15. Presence on non-participants | Was anyone else present besides the participants and researchers? | No, only BS, LF, and the five experts were present | - |
| 16. Description of sample | What are the important characteristics of the sample? E.g., demographic data, date | The experts were all experienced practitioners in the area of employee mental health programs, holding an academic degree in psychology and/or certifications in specific mental health professions, e.g., coach, counsellor; three of the experts were representatives of one of the selected EMHPs such that there was one representative for each of the three EMHPs; none of the experts was involved as an expert in the fourth iteration | Methods |
| *Data collection* | | | |
| 17. Interview guide | Were questions, prompts, guides provided by the authors? Was it pilot tested? | The focus group discussion followed a semi-structured guideline based on the objectives of the focus group, i.e., presentation and reflection of the taxonomy and application of the taxonomy to the selected EMHPs; all experts received a pre-read document stating the context and objectives of the focus group to ensure an efficient focus group session | - |
| 18. Repeat interviews | Were repeat interviews carried out? If yes, how many? | Not applicable | - |
| 19. Audio/visual recording | Did the research use audio or visual recording to collect the data? | The focus group session was audio recorded | - |
| 20. Field notes | Were field notes made during and/or after the interview or focus group? | Field notes were made on relevant input on the taxonomy and on the classification of one of the selected EMHPs during the focus group session; the audio recording was transcribed after the focus group session for analysis and documentation purposes | - |
| 21. Duration | What was the duration of the interviews or focus group? | The focus group session took 60 minutes | - |
| 22. Data saturation | Was data saturation discussed? | Data saturation was not discussed with participants | - |
| 23. Transcripts returned | Were transcripts returned to participants for comment and/or correction? | The transcript was not returned to participants; the participants received the taxonomy to independently complete the classifications of the remaining two EMHPs | - |
| **Domain 3: Analysis and findings** | | | |
| *Data analysis* | | | |
| 24. Number of data coders | How many data coders coded the data? | BS consolidated the classifications and conducted the qualitative and the interrater analysis based on the classifications of the focus group experts; LF supported the qualitative analysis and reviewed the interrater analysis | - |
| 25. Description of the coding tree | Did authors provide a description of the coding tree? | Not applicable | - |
| 26. Derivation of themes | Were themes identified in advance or derived from the data? | Not applicable | - |
| 27. Software | What software, if applicable, was used to manage the data? | Microsoft Word was used to create the transcript; Microsoft Excel was used to perform the interrater analysis | - |
| 28. Participant checking | Did participants provide feedback on the findings? | No | - |
| *Reporting* | | | |
| 29. Quotations presented | Were participant quotations presented to illustrate the themes/findings?  Was each quotation identified? E.g., participant number | No quotations were presented in the manuscript to support the findings | - |
| 30. Data and findings consistent | Was there consistency between the data presented and the findings? | Study findings, i.e., the classifications of the three selected EMHPs and the results of the qualitative and the interrater analysis (Multimedia Appendix 9) were reported such that they were consistent with the collected data, i.e., the concrete input of the experts during and after the focus group session | Results; Multimedia Appendix 9 |
| 31. Clarity of major themes | Were major themes clearly presented in the findings? | Not applicable | - |
| 32. Clarity of minor themes | Is there a description of diverse cases or discussion of minor themes? | Not applicable | - |

Based on: Tong A, Sainsbury P, Craig J; Consolidated criteria for reporting qualitative research (COREQ): a 32-item checklist for interviews and focus groups; Int J Qual Health Care 2007; 19(6): 349–357; doi: [10.1093/intghq/mzm042](https://doi.org/10.1093/intqhc/mzm042).
