# Supplementary material for: A Proposed Taxonomy to Holistically Classify Employee Mental Health Programs: Qualitative Taxonomy Development Study
Source: Interact J Med Res. 2025 Dec 18;14:e67752. doi: 10.2196/67752 (PMC12746229; doi:10.2196/67752)
Supplement: Checklist 6 [file ijmr-v14-e67752-s016.docx]

**Checklist 6. The 35-item ACCORD checklist for the focus group.**

| **No.** | **Item** | **Description** | **Item reporting** |
| --- | --- | --- | --- |
| **Section: Title** | | | |
| T1 | Identify the article as reporting a consensus exercise and state the consensus methods used in the title. | Only one part of the whole article, the taxonomy evaluation, comprised a consensus exercise, namely a focus group with qualitative and interrater analysis. | - |
| **Section: Introduction** | | | |
| I1 | Explain why a consensus exercise was chosen over other approaches. | A focus group with qualitative and interrater analysis was chosen as it represents an established method for taxonomy evaluation. | Methods |
| I2 | State the aim of the consensus exercise, including its intended audience and geographical scope (national, regional, global). | The aim of the focus group was to reach consensus between the five focus group experts on the subjective ending conditions of the taxonomy to be able to conclude that the final taxonomy was concise, robust, comprehensive, extendible, and explanatory. Further, the aim was to achieve the three evaluation goals, confirming that the taxonomy can be used to describe, classify, and analyze EMHPs. Consensus was measured through the interrater analysis. The five focus group experts were German nationals and EMHP specialists with practitioner background, with some of them additionally having a scientific background. | Methods |
| I3 | If the consensus exercise is an update of an existing document, state why an update is needed, and provide the citation for the original document. | Not applicable | - |
| **Section: Methods** | | | |
| *Registration* | | | |
| M1 | If the study or study protocol was prospectively registered, state the registration platform and provide a link. If the exercise was not registered, this should be stated. | The study was not registered. The ethics committee of Witten/Herdecke University reviewed and approved this research project without raising any ethical concerns (S-317/2023). | Methods |
| *Selection of SC and/or panellists* | | | |
| M2 | Describe the role(s) and areas of expertise or experience of those directing the consensus exercise. | BS moderated the focus group and conducted the qualitative and the interrater analysis. LF co-moderated the focus group and supported the qualitative analysis and reviewed the interrater analysis. Both moderators conduct research in the digital health discipline. BS has a business background and his research focuses on digital and mental health and the intersection. LF has a medical background, is a medical doctor, and his research focuses on AI in health care, digital tools and delivery, and (digital) mental health. | - |
| M3 | Explain the criteria for panellist inclusion and the rationale for panellist numbers. State who was responsible for panellist selection. | The experts were selected such that all participants were EMHP specialists with practitioner background and none of them had been involved in the fourth iteration. One representative of each of the three classified EMHPs participated to ensure adequate program-specific insight. The experts were identified through purposive sampling, targeting a number of four to six experts with ultimately five experts participating, a reasonable focus group size and in line with focus groups conducted by other researchers who published their work. BS was primarily responsible for the expert selection. | Methods |
| M4 | Describe the recruitment process (how panellists were invited to participate). | Experts were contacted via email or LinkedIn. The initial message contained a one-pager with the most relevant information on the focus group. One of the experts was part of the broader network of the authors. No relationship to the other experts was established prior to the study. Some contacted experts refused to participate in the focus group due to lack of time. | Methods |
| M5 | Describe the role of any members of the public, patients or carers in the different steps of the study. | The focus group did not involve members from the public nor patients. Focus group experts consisted of representatives of providers and general EMHP experts. | Methods |
| *Preparatory research* | | | |
| M6 | Describe how information was obtained prior to generating items or other materials used during the consensus exercise. | Information for creation of the taxonomy prior to taxonomy evaluation through the focus group was collected through two scoping reviews and two interview studies, which together formed the core of the taxonomy development process. The whole taxonomy development process is described in detail in the Methods section of the article. | Methods |
| M7 | Describe any systematic literature search in detail, including the search strategy and dates of search or the citation if published already. | All details on the two scoping reviews are provided in the Methods section of the article and in the respective Multimedia Appendices 1-4 and in the Checklists 1-2. | Methods; Multimedia Appendices 1-4; Checklists 1-2 |
| M8 | Describe how any existing scientific evidence was summarised and if this evidence was provided to the panellists. | Existing taxonomies and further research on EMHPs and general mental health interventions were considered to develop the new EMHP taxonomy. The focus group experts were provided with the final taxonomy version and most relevant insights from the fourth iteration were shared during the focus group discussion. | - |
| *Assessing consensus* | | | |
| M9 | Describe the methods used and steps taken to gather panellist input and reach consensus (for example, Delphi, RAND-UCLA, nominal group technique). | The focus group experts jointly reflected on the taxonomy and individually applied it to three selected EMHPs, and the discussion and the classifications were subsequently analyzed. The reflection was qualitatively analyzed based on explicit expert input. For the analysis of the classifications, the interrater analysis was used, determining proportion of observed agreement and Fleiss kappa. | Methods |
| M10 | Describe how each question or statement was presented and the response options. State whether panellists were able to or required to explain their responses, and whether they could propose new items. | For the reflection, the taxonomy was presented to the focus group experts by dimension and every dimension was individually discussed. For the classifications, the taxonomy as a whole was presented to the experts. Each expert was required to select the one or several characteristics per dimension, which they deemed suitable, for each of the three EMHPs, to classify the EMHPs. The first EMHP was classified during the focus group session, the other two EMHPs were classified afterwards by each of the experts. The remaining two classifications were shared back to the authors for analysis. | Methods |
| M11 | State the objective of each consensus step. | Objective of the focus group discussion was to reach consensus on the five subjective ending conditions. Objective of the EMHP classification was to receive data that could be used to conduct the interrater analysis to derive the proportion of observed agreement and Fleiss kappa, confirming that the three evaluation goals were achieved. | Methods |
| M12 | State the definition of consensus (for example, number, percentage, or categorical rating, such as ‘agree’ or ‘strongly agree’) and explain the rationale for that definition. | Consensus on the five subjective ending conditions was defined as all experts agreeing on the form of the taxonomy (as verbally stated during the focus group session) and being able to classify the EMHPs after discussing the taxonomy (as confirmed through the resulting classifications). Consensus on the classifications was measured through overall proportion of observed agreement and Fleiss kappa with interpretation thresholds used as defined by literature. | Methods; Results |
| M13 | State whether items that met the prespecified definition of consensus were included in any subsequent voting rounds. | Not applicable | - |
| M14 | For each step, describe how responses were collected, and whether responses were collected in a group setting or individually. | Field notes on relevant input on the reflection and discussion were taken by the moderators and the audio recording was transcribed after the focus group session for analysis and documentation purposes. Application of the taxonomy to the first EMHP happened during the focus group session, but experts were asked to classify the EMHP individually. Experts annotated the characteristics they would select per dimension through the Zoom annotate function. The answers were collected by the moderators. The other two EMHPs were classified by each focus group expert individually and offline in written form after the focus group session by marking each selected characteristic for each of the two EMHPs. The offline classifications were sent back to the authors. The authors collected the data to perform the interrater analysis. | Methods |
| M15 | Describe how responses were processed and/or synthesised. | The field notes and the transcript were reviewed after the session to qualitatively analyze whether the subjective ending conditions were met based on explicit expert statements and whether the experts were able to use the taxonomy to classify EMHPs based on the classification of the first EMHP and the subsequent group discussion. The classification data were organized such that the interrater analysis could be conducted. Every characteristic of every dimension, per each focus group expert and per EMHP, was binary coded, i.e., whether it was selected or not. Based on this binary coding, the interrater analysis was conducted. The proportion of observed agreement and Fleiss kappa were calculated per dimension and overall. Microsoft Excel was used to conduct the analysis. | Methods |
| M16 | Describe any piloting of the study materials and/or survey instruments. | No pilot was conducted. The focus group was well prepared together with other researchers who had conducted focus groups before. | - |
| M17 | If applicable, describe how feedback was provided to panellists at the end of each consensus step or meeting. | The focus group experts saw the classifications of the first EMHP. Questions were asked to the experts to understand the reasons for choosing the selected characteristics in cases where there were differences in the classifications. | - |
| M18 | State whether anonymity was planned in the study design. Explain where and to whom it was applied and what methods were used to guarantee anonymity. | There was no anonymity given nor planned between the focus group experts given the focus group was conducted as an open discussion through a live video call. To protect the privacy of the focus group experts, all of them were provided with a privacy statement and signed a consent form prior to the focus group session, stating that they would keep the names of the other focus group experts confidential outside of this group. | - |
| M19 | State if the steering committee was involved in the decisions made by the consensus panel. | The moderators of the focus group did not classify the three EMHPs. The moderators were involved in the discussion for providing context and insights from the fourth iteration of the taxonomy development process to answer questions from the focus group experts on the taxonomy and to provide more clarity on the dimensions and characteristics. | - |
| *Participation* | | | |
| M20 | Describe any incentives used to encourage responses or participation in the consensus process. | The focus group experts did not receive compensation for their participation. A potential benefit for the focus group experts was to be connected with other experts in this domain through the focus group. | - |
| M21 | Describe any adaptations to make the surveys/meetings more accessible. | The focus group session was conducted in German language as all participants were fluent in German. The taxonomy was presented in English language; all experts were able to read and understand the English terms. | - |
| **Section: Results** | | | |
| R1 | State when the consensus exercise was conducted. List the date of initiation and the time taken to complete each consensus step, analysis, and any extensions or delays in the analysis. | The focus group session was planned during March/April 2024. The focus group session took place on 16 April 2024 and took 60 minutes. The classifications of the two remaining EMHPs were all collected by mid of May 2025. The interrater analysis was conducted after having received all data. | - |
| R2 | Explain any deviations from the study protocol, and why these were necessary. | Not applicable | - |
| R3 | For each step, report quantitative (number of panellists, response rate) and qualitative (relevant socio-demographics) data to describe the participating panellists. | Five experts participated in the focus group. The experts were all experienced practitioners in the area of employee mental health programs, holding an academic degree in psychology and/or certifications in specific mental health professions, e.g., coach, counsellor. Three of the experts were representatives of one of the selected EMHPs such that there was one representative for each of the three EMHPs. None of the experts was involved as an expert in the fourth iteration. All five experts provided the offline classifications of the two remaining EMHPs after the focus group session. | Methods; Results |
| R4 | Report the final outcome of the consensus process as qualitative (for example, aggregated themes from comments) and/or quantitative (for example, summary statistics, score means, medians and/or ranges) data. | The qualitative analysis, based on explicit expert input, supported the conclusion that the taxonomy was concise, robust, comprehensive, extendible, and explanatory. The interrater analysis yielded an overall proportion of observed agreement of 85% and Fleiss kappa of 66%. Proportion of observed agreement ranged from 64% to 100% and Fleiss kappa ranged from 20% to 100% across dimensions. The results were statistically significant with *P* values ranging from *P*=.004 to *P*<.001. The consensual understanding and reflection of the taxonomy during the focus group session and the successful classification of the 3 EMHPs with high overall agreement measures reconfirmed that the subjective ending conditions were met. Further, the classifications demonstrated that the three evaluation goals were achieved. | Results |
| R5 | List any items or topics that were modified or removed during the consensus process. Include why and when in the process they were modified or removed. | Not applicable | - |
| **Section: Discussion** | | | |
| D1 | Discuss the methodological strengths and limitations of the consensus exercise. | The selected method focus group represents an established method for taxonomy evaluation as proposed by Szopinski et al. (Szopinski et al., 2019, Because Your Taxonomy is Worth IT: Towards a Framework for Taxonomy Evaluation) and has been used by several researchers who developed and evaluated taxonomies. The set of focus group experts is solid with participants, who were all experienced practitioners in the area of employee mental health programs, holding an academic degree in psychology and/or certifications in specific mental health professions, e.g., coach, counsellor, while some additionally having other academic and/or professional backgrounds with different depth of scientific experience. The deep expertise and diverse experiences ensured consideration of different perspectives. Additional validity of the taxonomy evaluation stems from the fact that none of the focus group experts was involved in the taxonomy development process. A potential limitation of the set of focus group experts is that only experts from Germany or mainly with practical experience in Germany were included in the focus group. This might limit the strength of the taxonomy evaluation regarding the consideration of international perspectives. However, this only concerns the evaluation. To ensure general representativeness and universal applicability of the taxonomy, also regarding geographical diversity, we ensured a diverse set of experts in the fourth iteration with experts from 7 different countries across 3 different continents. | Methods; Discussion |
| D2 | Discuss whether the recommendations are consistent with any pre-existing literature and, if not, propose reasons why this process may have arrived at alternative conclusions. | Not applicable | - |
| **Section: Other information** | | | |
| O1 | List any endorsing organisations involved and their role. | Not applicable | - |
| O2 | State any potential conflicts of interests, including among those directing the consensus study and panellists. Describe how conflicts of interest were managed. | Conflicts of interest may have come from the fact that the five focus group experts included representatives of the providers of the three EMHPs that were classified. To manage conflicts of interest, the EMHPs were classified based on an outside-in perspective, i.e., information that could be obtained publicly by any individual, for instance via the respective provider’s website. Provider representatives had their own one independent vote in the consensus exercise like any other focus group expert. For the classification of the first EMHP, the respective provider representative did not have the right to change selections made by other experts. However, the expert could answer questions from the moderators or other experts regarding factual characteristics of the EMHP where necessary. The classifications of the two remaining EMHPs were collected offline, automatically avoiding any potential influence of a provider representative. By this, the potential conflict of interest was adequately managed. | - |
| O3 | State any funding received and the role of the funder. | Not applicable | - |

Based on: Gattrell WT, Logullo P, van Zuuren EJ, et al; ACCORD (ACcurate COnsensus Reporting Document): A reporting guideline for consensus methods in biomedicine developed via a modified Delphi; PLOS Med 2024; 21(1):e1004326; doi: [10.1371/journal.pmed.1004326](https://doi.org/10.1371/journal.pmed.1004326).
